# Metal Nitride Catalysts for Photoelectrochemical and Electrochemical Catalysis

**DOI:** 10.1002/EXP.20240013

**Published:** 2025-03-02

**Authors:** Hee Ryeong Kwon, Jin Wook Yang, Ho Won Jang

**Affiliations:** ^1^ Department of Materials Science and Engineering Research Institute of Advanced Materials Seoul National University Seoul Republic of Korea; ^2^ Department of Chemical and Biomolecular Engineering University of California Berkeley California United States; ^3^ Advanced Institute of Convergence Technology Seoul National University Suwon Republic of Korea

**Keywords:** electrocatalysis, hydrogen evolution, metal nitrides, oxygen evolution, photoelectrocatalysis

## Abstract

Metal nitrides have emerged as promising materials for photoelectrochemical and electrochemical catalysis due to their unique electronic properties and structural versatility, offering high electrical conductivity and abundant active sites for catalytic reactions. Herein, we comprehensively explore the characteristics, synthesis, and application of diverse metal nitride catalysts. Fundamental features and catalytic advantages of metal nitrides are presented in terms of electronic structure and surface chemistry. We deal with synthetic principles and parameters of metal nitride catalysts in terms of nitrogen source, introducing synthesis strategies of metal nitrides with various morphologies and phases. Recent progress of metal nitride catalysts in (photo)electrochemical reactions, such as hydrogen evolution, oxygen evolution, oxygen reduction, nitrogen reduction, carbon dioxide reduction, and biomass valorization reactions, is discussed with their tailored roles. By providing future direction for remaining challenges, this review aims to guide the design of metal nitride catalysts from a materials point of view, contributing to expanding into energy and environmental technologies.

## Graphical abstract

Metal nitrides exhibit excellent electrical conductivity and catalytic activity, making them promising for (photo)electrochemical reactions. This review explores their electronic structure, surface chemistry, and synthesis strategies, emphasizing morphology and phase control. Detailed discussions on catalyst designs for hydrogen/oxygen evolution, oxygen/nitrogen/carbon dioxide reduction, and biomass valorization reactions provide significant insights for optimizing performance.

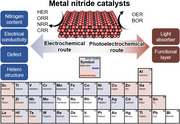

## 1 Introduction

Developing effective energy conversion technologies has become an imminent task due to the world's rising energy consumption and growing environmental issues. In this context, electrocatalysis combined with renewable energy, which can produce sustainable energy sources and chemical fuels without carbon emissions, has gained significant attention in energy conversion technologies. Substantial progress has also been made in various electrochemical reactions that use abundant resources such as H_2_O, N_2_, O_2_, CO_2_, and biomass byproducts as reactants to generate high‐value‐added chemical energy [1]. Diverse catalyst materials—including metals, oxides, hydroxides, nitrides, sulfides, and carbides—have been developed to enhance the kinetics of these reactions [2, 3]. Nevertheless, practical applications still require validation of catalyst materials for economic feasibility, operational stability, and scalability, along with a thorough understanding of catalyst behavior.

Metal nitrides have emerged as promising materials in (photo)electrocatalysis due to their unique electronic structure and catalytic properties [4, 5, 6]. Their distinctive electronic features, characterized by a combination of metallic conductivity and inherent catalytic activity, make them highly desirable for replacing noble metal‐based catalysts in electrocatalytic applications. Moreover, controlled synthesis of metal nitrides enables tuning their catalytic activity towards specific reactions, enhancing efficiency and durability in electrochemical processes. By identifying active sites resulting from interactions between metals and nitrogen, researchers have elucidated the relationship between structure and activity, guiding the design of nitride electrocatalysts [7]. In addition, metal nitride semiconductors have been mainly exploited as light absorbers with high light absorption efficiency in photoelectrochemical reactions.

This review explores the advancement of metal nitrides for (photo)electrocatalysis, focusing specifically on transition metal nitrides and III‐nitrides. We begin with an overview of metal nitrides’ crystal phases and electrical properties and discuss the advantages of catalytic behavior arising from these features. The following section describes the synthesis strategies of metal nitrides, categorizing them into direct ammonolysis, indirect ammonolysis, solution process, and vapor deposition. We examine the formation mechanisms of metal nitrides through various synthetic examples that control the phases, compositions, and structures. Furthermore, the structure‐activity relationships and development strategies of metal nitrides are covered in detail for various electrochemical reactions, including hydrogen evolution reaction (HER), oxygen evolution reaction (OER), oxygen reduction reaction (ORR), nitrogen reduction reaction (NRR), carbon dioxide reduction reaction (CRR), and biomass oxidation reaction (BOR). Applications in photoelectrochemical reactions are also discussed, with a focus on metal nitride semiconductors. Finally, we propose development directions of metal nitrides for achieving competitive (photo)electrocatalysis.

## 2 Metal Nitrides

### 2.1 Fundamental Features

**FIGURE 1:**
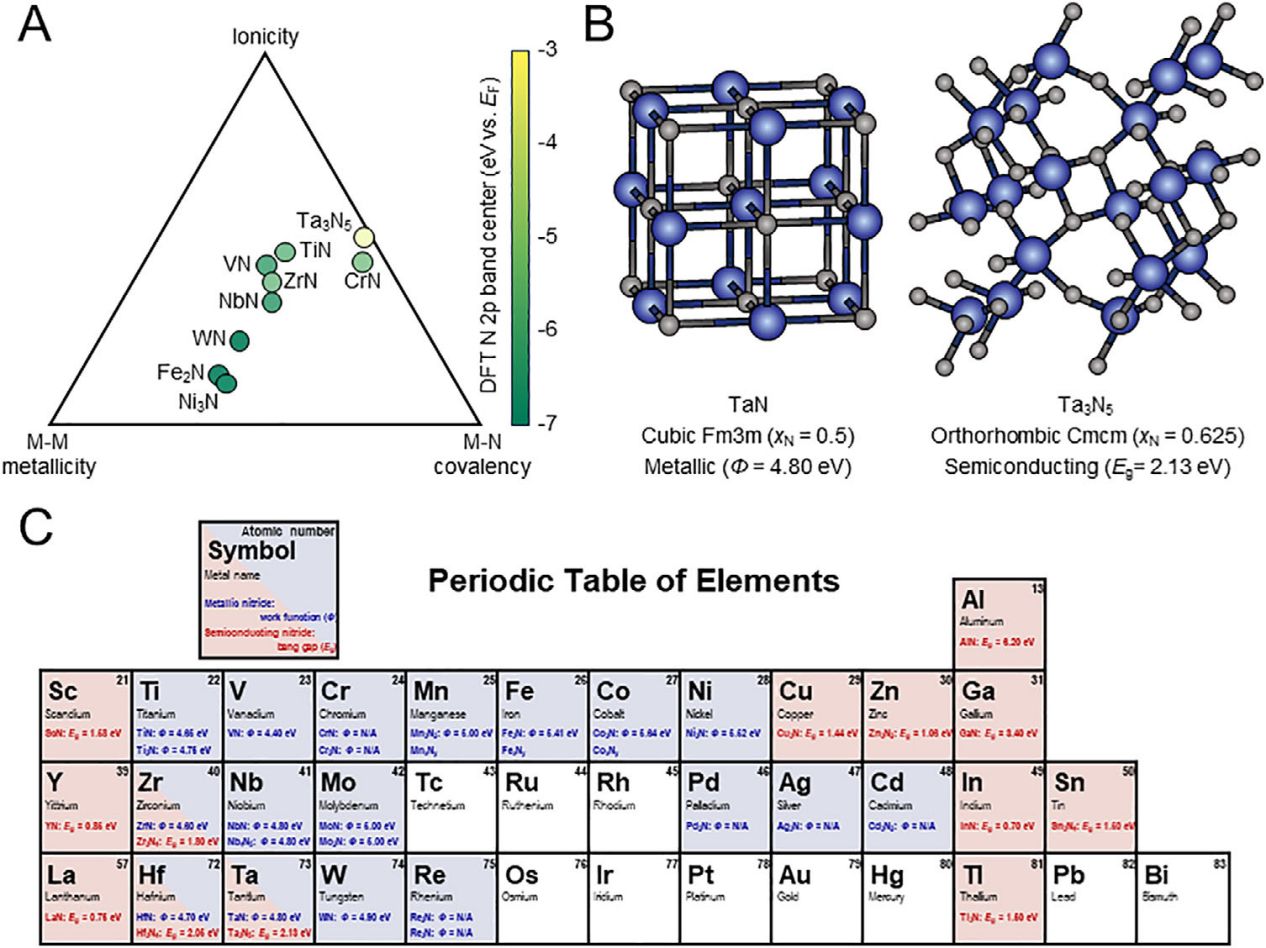
(A) Metallicity, ionicity, and covalency of metal nitrides plotted on the van Arkel–Ketelaar diagram based on the DFT‐computed electron density. Reprinted with permission [13]. Copyright 2023, Cell Press. (B) Crystal structures of TaN and Ta_3_N_5_. (C) Periodic table of elements for metal nitrides.

Metal nitrides are compounds that consist of nitrogen bonded to metallic elements, and their properties vary depending on the interaction between metal atoms and nitrogen atoms. Generally, it is classified into ionic, covalent, and metallic characteristics according to the bonding type, but most metal nitrides exhibit mixed characteristics of ionicity, covalency, and metallicity [8]. Ionic metal nitrides generally form between highly electropositive metals (e.g., alkali or alkaline earth metals) and nitrogen. These compounds tend to have high lattice energies and possess high reactivity. Since ionic metal nitrides directly react with water through hydrolysis to generate ammonia (e.g., Li_3_N + 3H_2_O → 3LiOH + NH_3_), they cannot be used as catalysts but only to synthesize metal nitrides. So, in this review, we mainly deal with transition metal nitrides (IIIb‐IIb groups) and III‐nitrides for (photo)electrocatalysis. These metal nitrides are interstitial compounds, where the nitrogen atoms are integrated into the interstitial sites of the parent metals, exhibiting covalent compounds, ionic crystals, and metallic natures [4]. The metal−nitrogen (M−N) bonding expands the parent metal lattice and manipulates the *d*‐band structure of parent metals [9, 10, 11]. In more detail, their *d*‐band occupation shrinks, and the density of states near the Fermi level increases. The resulting increase in valence electrons imparts the electrical conductivity and catalytic activity of metal nitrides [5]. Besides, the strong M−N bonding prevents the nitride ion (N^3−^) from being substituted, leading to the high chemical stability of metal nitrides [12]. The electrical conductivity of metal nitrides has a large correlation with the nitrogen 2*p*‐band center relative to the Fermi level. Based on density functional theory (DFT) calculations and X‐ray emission spectroscopy, Peng et al. have revealed the relationship between the nitrogen 2*p*‐band center and the bonding characteristics of metal nitrides [13]. Lowering the nitrogen 2*p*‐band center relative to the Fermi level weakens the M−N bonds, increasing metal−metal (M−M) interactions in nitrides. The weakening of M−N bonding by the relative distance from the Fermi level and nitrogen 2*p*‐band center is also described by the orbital Hamilton population (COHP) analysis, which indicates orbital overlaps. As shown in Figure 1A, the resulting van Arkel–Ketelaar diagram shows the metallicity, ionicity, and covalency of metal nitrides. Fe_2_N and Ni_3_N, featuring the lowest nitrogen 2*p*‐band center, have the weakest M−N bonds and the most occupied antibonding M−N orbitals in COHP. Furthermore, Sun et al. completed the van Arkel–Ketelaar diagrams of ternary metal nitrides using DFT calculations [14]. The strong M−M bonds enhance the electrical conductivity, but the catalytic activity is determined by a complex effect of not only electrical conductivity but also other factors, such as active sites, nitrogen contents, and phases.

Metal nitrides have a variety of crystal structures depending on the stoichiometry of the compound, and the crystal structure significantly influences the electronic properties of metal nitrides. Generally, transition metal nitrides exhibit rock salt (e.g., TiN, ZrN, NbN, TaN), hexagonal closed‐packed (e.g., ScN), tetragonal (e.g., Ti_2_N), and simple hexagonal (e.g., MoN, WN) structures. Also, III‐nitrides have wurtzite structures (e.g., AlN, GaN, InN). Furthermore, in the ternary system, the ABN_3_‐type perovskite structure (e.g., LaTiN_3_) and antiperovskite structure (e.g., Fe_3_NiN, Cu_3_PdN), where the positions of metal and nitrogen are inverted, are also observed. Since the nitrogen content inside the lattice is changed depending on the crystal structure, the electronic structure varies even in the same M−N system. Tantalum nitride, which shows a variety of nitrogen contents from Ta_2_N to Ta_3_N_5_, is the most obvious example [15]. Figure 1B shows the different crystal structures of representative tantalum nitride: metallic TaN (cubic Fm3m) and semiconducting Ta_3_N_5_ (orthorhombic Cmcm). The nitrogen content of orthorhombic Ta_3_N_5_ (*x*_N_ = 0.625) is higher than that of cubic TaN (*x*_N_ = 0.5) [16]. As the nitrogen content increases, the filled states of the *d*‐band become narrower as the *d*‐band electrons combine with nitrogen [17]. In contrast, the unfilled states above the Fermi level become wider, changing the electrical conductivity of tantalum nitrides. Further increases in the nitrogen content make the energy level of unoccupied states higher and occupied states lower, forming the energy difference between the valence and conduction bands. Regarding the aforementioned M−N bondings, the expansion of the nitrogen 2*p*‐band strengthens the covalency of M−N bonds through interaction with the Ta 5*d*‐band, reducing the M−M interactions. As a result, more electrons in Ta_3_N_5_ stay in a specific energy level, forming a band gap of 2.13 eV and semiconducting features [18]. Based on previous reports, we organized metal nitrides with covalency and metallicity into a periodic table, as shown in Figure 1C. Representative phases of each metal nitride were presented along with their work function and band gap according to their metallic and semiconducting natures.

### 2.2 Catalytic Advantages

**FIGURE 2:**
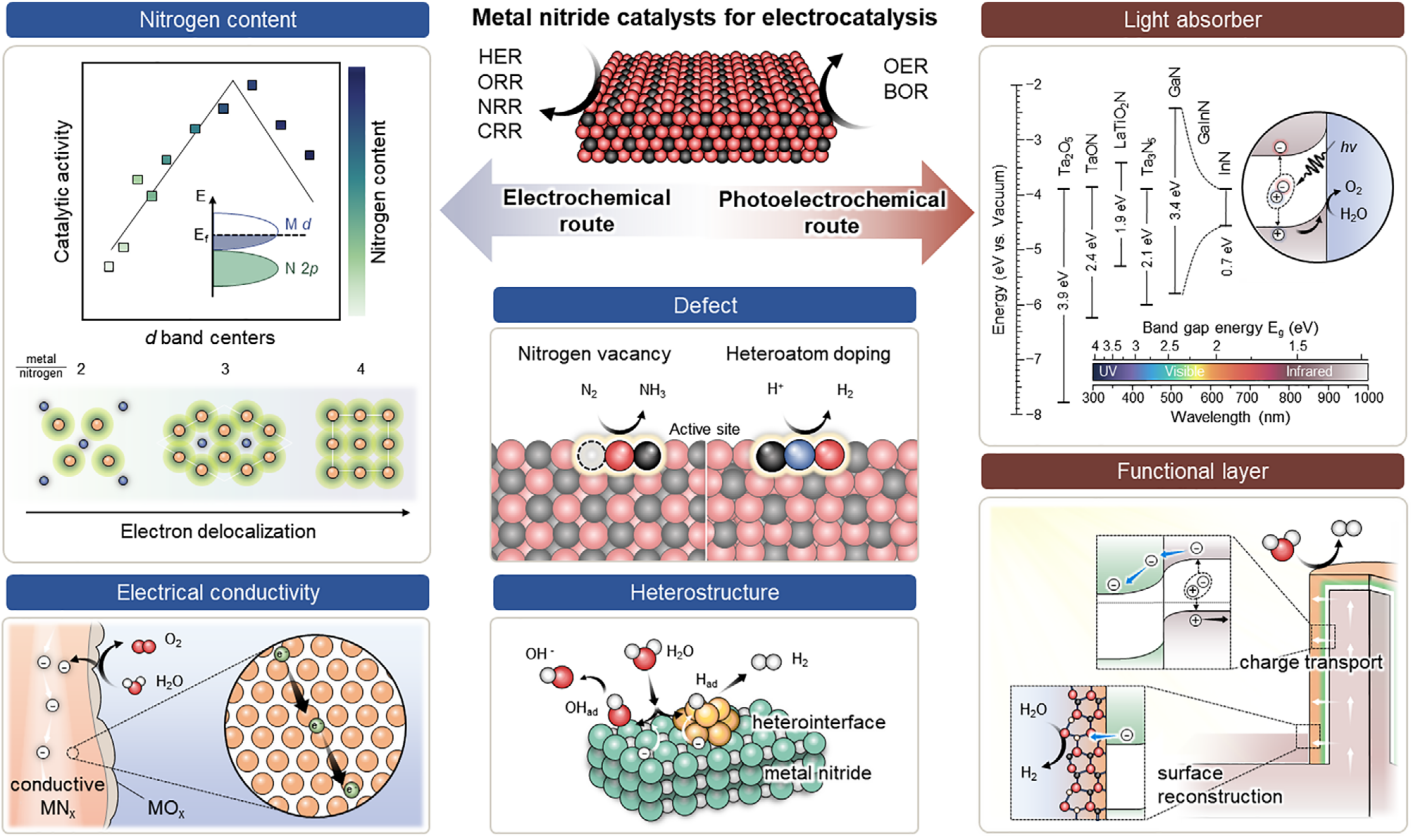
Catalytic advantages of metal nitrides.

Metal nitrides have been extensively utilized in (photo)electrocatalysis due to their unique advantages, as illustrated in Figure 2. In electrocatalysis, the distinctive catalytic activity of metal nitrides arises from the electronic interaction between metal and nitrogen. Manipulating the electronic structure through variations in crystal phase and nitrogen content allows metal nitrides to develop many inherent advantages.

One key advantage is their high electrical conductivity, which reduces charge transfer resistance and enhances overall catalytic efficiency. Most metal nitride electrocatalysts exhibit metallicity, resulting in significantly lower electrical resistance compared to compounds like oxides, sulfides, phosphates, carbides, and borides. Specifically, this metallicity arises from the hybrid bonding nature of metal nitrides, where both covalent and ionic bonds coexist between metal atoms and nitrogen atoms. Consequently, conductive nitrides can be used both as standalone electrodes and as scaffolds supporting other catalysts. While electrical conductivity generally increases with the nitrogen content, it alone does not determine catalytic activity [19]. Therefore, optimizing the nitrogen content in metal nitrides is crucial to balancing both conductivity and active sites for catalysis.

Another advantage is the ability to effectively control the adsorption energy of reaction intermediates through the formation of M−N bonding. Namely, the unique electronic structure of metal nitrides compared to oxide, sulfides, phosphates, carbides, and borides is characterized by a moderate *d*‐band center and strong M−N bonding, which allows for more precise control of the adsorption and desorption of reaction intermediates. The incorporation of nitrogen into the metal lattice modifies the *d*‐band electron configuration, creating a Pt‐like *d*‐band structure around the Fermi level [4]. This alteration, combined with charge redistribution between electronegative nitrogen and the metal, influences electron‐donating properties and modulates the degree of adsorption with intermediates. This tuning of intermediate binding energies results in higher catalytic activity and selectivity compared to oxides, which can bind intermediates too strongly, and sulfides, which may have weaker interactions.

Furthermore, the corrosion resistance and chemical stability of metal nitrides improve the durability of electrocatalysts. Nitrogen incorporation prevents metal dissolution and increases the metal's valence state. N‐rich phases, in particular, exhibit increased valence states that suppress oxidation, enabling stable and active catalysis [20]. Alternatively, a thin oxide or hydroxide layer that forms on the metal nitride surface can protect the nitride phase. Under oxidizing potentials, surface oxidation inevitably occurs, but this reconstruction can provide a new activated surface and a conductive nitride core. Hence, the stability of metal nitrides can be enhanced through understanding the operating environment and designing accordingly.

Moreover, various engineering strategies can further improve the catalytic activity of metal nitrides. Composition and defect engineering induce redistribution of the electronic structure, finely tuning the adsorption of intermediates to boost catalytic activity. Precise adjustment of the metal‐to‐nitrogen ratio, doping, and vacancy results in balanced catalysts with improved conductivity, active sites, and structural stability. Specifically, this adjustment affects the *d*‐band center and Fermi level, thus directly impacting the catalyst's conductivity, charge transfer ability, and overall activity. Moreover, doping with foreign elements can introduce new active sites or modify existing ones, thereby increasing the density of catalytic sites and enhancing electron mobility. The introduction of vacancies and defects further modulates the local electronic environment, allowing for optimized activation energies and improved mass transport of reactants. Additionally, heterostructure engineering creates interfaces with various materials such as metals, oxides, sulfides, and carbides, facilitating effective reaction pathways in complex catalytic reactions [21]. The heterointerfaces can alter the local electronic density and induce strain effects, improving the adsorption and desorption rates of intermediates. This is particularly advantageous in complex catalytic reactions with multi‐step processes, such as those found in nitrogen and carbon dioxide reduction reactions. These approaches optimize the catalytic performance of metal nitrides, offering versatile solutions for advancing catalysis in diverse electrochemical reactions.

In photoelectrocatalysis, transition metal‐based (oxy)nitrides and III‐nitrides with semiconducting phases have been mainly used. Their major advantage is the visible light active adsorption region. A representative transition metal nitride semiconductor, Ta_3_N_5_, has a light absorption range below about 600 nm. InGaN of III‐nitrides has an absorption range below about 700 nm through alloy composition control. Thus, metal nitrides can utilize the visible light range, which was limited in oxide‐based light absorbers such as TiO_2_, WO_3_, and BiVO_4_. Additionally, in transition metal‐based nitrides, narrow bandgap semiconductor phases have been newly reported through alloy composition control, indicating significant potential for developing nitride‐based light absorbers [22]. Another advantage of nitride semiconductors is that structural and morphological engineering can enhance light absorption efficiency and charge transport efficiency. Controlled crystal facets in nitrides facilitate efficient charge transport and separation, while doping or defect engineering suppresses charge recombination centers, improving charge transfer efficiency. Furthermore, nanostructures simultaneously increase light absorption efficiency and transport efficiency.

## 3 Synthetic Strategies of Metal Nitrides

The stable nature of nitrogen in the atmosphere prevents metal nitrides from forming naturally, unlike metal oxides. Accordingly, various synthesis processes must be performed to obtain metal nitrides, and each method has distinct advantages and limitations. The synthesis methods are classified according to the origin of the nitrogen atom that forms a bond with the host metal: ammonia gas, nitrogen gas, and powder or solution containing N species. Hence, depending on nitrogen sources, the heat treatment is classified into the nitridation process itself or crystallization of the already‐formed nitride phases. This section mainly deals with various synthetic strategies of metal nitrides and focuses on their phases or morphological aspects. By examining the principles of each method, the optimal approaches for producing metal nitrides are introduced along with representative reports.

### 3.1 Direct Ammonolysis

**FIGURE 3:**
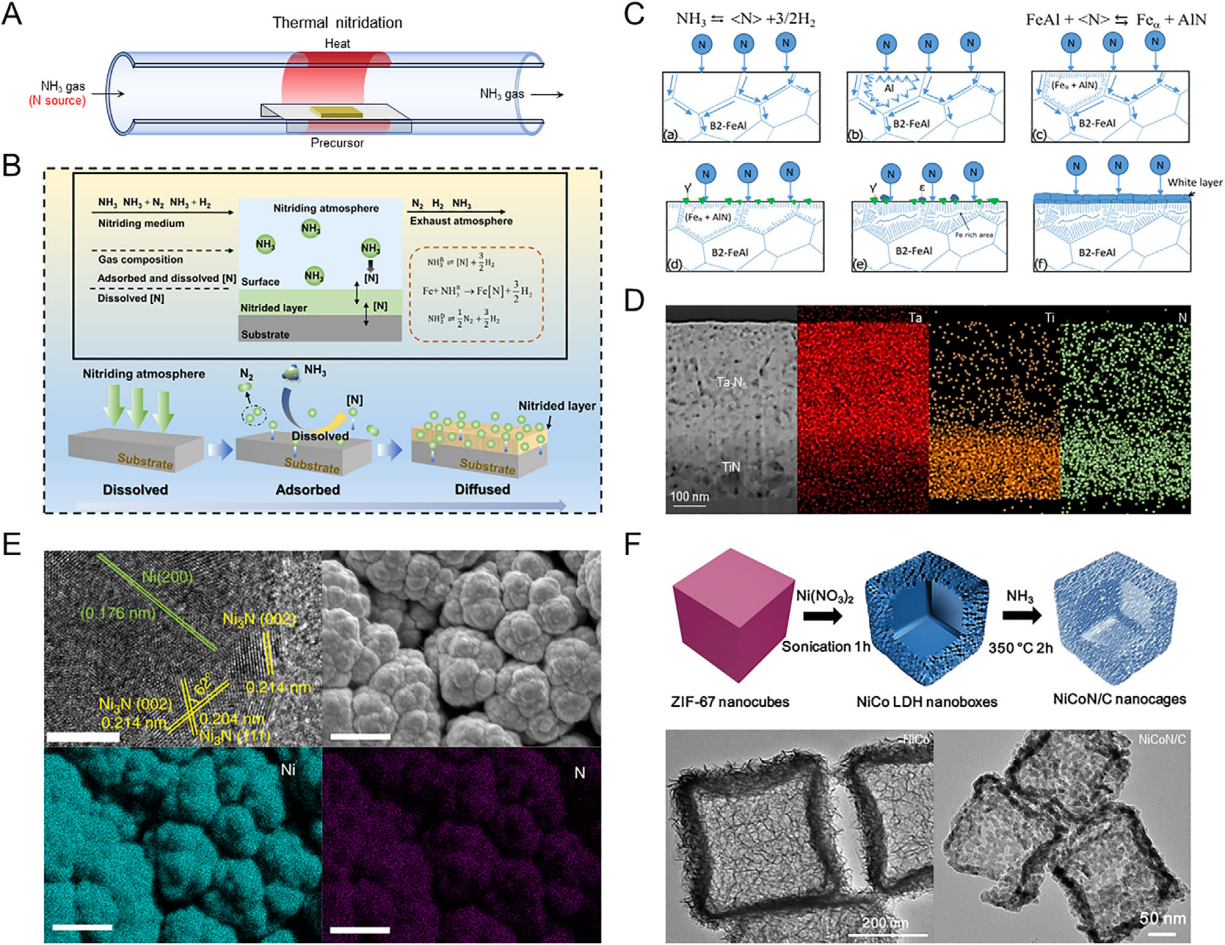
(A) Schematic illustration of the direct ammonolysis. (B) Interface reaction process of gas nitridation. Reprinted with permission [23]. Copyright 2023, MDPI. (C) A mechanism of the effect of the nitriding potential K_N_ on the ‘‘white layer’’ formation. Reprinted with permission [28]. Copyright 2015, Elsevier. (D) Cross‐sectional STEM image and EDX mapping of Ta_3_N_5_/TiN. Reprinted with permission [30]. Copyright 2024, Wiley‐VCH GmbH. (E) HRTEM image of Ni_3_N/Ni interface (scale bar, 5 nm), SEM image, and EDX mapping of Ni_3_N/Ni/NF (scale bar, 500 nm). Reprinted with permission [32]. Copyright 2018, Nature Publishing Group. (F) A formation process of strongly coupled NiCoN/C nanocages and HRTEM images of NiCo and NiCoN/C. Reprinted with permission [38]. Copyright 2019, Wiley‐VCH GmbH.

Direct ammonolysis is the most representative method for synthesizing metal nitrides, leveraging the reactivity of ammonia as a nitrogen source. This process involves the direct reaction of a metal or metal compound with ammonia gas at elevated temperatures. The general reaction can be represented as follows:

$$MO_{x}+NH_{3}\left(g\right)\to MN_{x}+H_{2}\left(g\right)$$

$$MO_{x}+NH_{3}\left(g\right)\to MN_{x}+H_{2}O\left(g\right)$$

where M, MO*_x_*, and MN*_x_* are metal, metal oxide, and metal nitrides. H_2_ or H_2_O gases are produced as byproducts. The process of direct ammonolysis is schematically illustrated in Figure 3A. The metal precursor can be in the form of pure metal, metal (hydro)oxide, or metal chloride. The process variables of nitridation include the ammonia flow rate, heating temperature, and reaction time. Ammonia gas is introduced into the reaction chamber containing the metal compound precursor. The flow rate of ammonia is controlled to ensure a sufficient supply of nitrogen throughout the reaction. The reaction chamber is heated to a specific temperature, depending on the reactivity of the metal compound and the target phase of the metal nitride. The high temperature facilitates the dissociation of ammonia and the subsequent formation of metal nitride. The reaction time is optimized to ensure complete conversion of the metal compound precursor to the nitride phase. Figure 3B shows the interface reaction process of direct ammonolysis [23]. The process consists of three steps: decomposition of ammonia, adsorption of active nitrogen atoms, and diffusion of active nitrogen atoms [24]. The flowed ammonia gas is thermally decomposed to the active nitrogen atoms, which adsorb and diffuse into the precursor. Reacting with active nitrogen atoms, the nitride layer becomes thicker above the precursor [25]. In the case of the alloy precursor, the nitride has a layered structure depending on the limited solubility of the nitrogen atom and the resulting nitriding potential (*K*_N_) difference [26]. Zhang and Le suggested a mechanism for the effect of the nitriding potential in a FeAl system, as shown in Figure 3C [27, 28]. When active nitrogen atoms diffuse into the grain boundary of the FeAl system, Al, which has a higher chemical affinity to nitrogen, diffuses into the grain boundary to form AlN outside of Fe. Depending on the *K*_N_ of Fe, nitrogen atoms diffuse into a deeper region to form the iron nitrides, contributing to the formation of a diffusion zone in the nitride layer. As *K*_N_ increases, the proportion of the iron nitride phase on the surface increases, and the layered structure of AlN and Fe decreases, forming a white layer on the surface.

Direct ammonolysis has the advantage of producing high‐purity metal nitrides because it uses high‐concentration ammonia and is performed in a controlled environment [29]. Furthermore, various metal compounds can be used as precursors, which is advantageous in designing catalysts. However, direct ammonolysis typically requires high temperatures (often above 700°C) to achieve complete conversion. Also, since ammonia is a toxic and corrosive gas, containing ammonia requires specialized equipment and procedures, increasing the complexity and cost of the synthesis process. Yang et al. replaced the previously used Ta foil with TiN film for the bottom electrode of the Ta_3_N_5_ photoelectrode [30]. The e‐beam evaporated Ti and Ta_2_O_5_ were used as precursors, and sufficient reaction at a high temperature of 950°C and high‐purity ammonia contributed to forming a highly crystalline Ta_3_N_5_/TiN layer. As shown in the scanning transmission electron microscope (STEM) image and energy dispersive X‐ray (EDX) mapping (Figure 3D), the diffusion of nitrogen atoms and complete nitridation were achieved regardless of the precursor type. The optimized nitridation condition can construct the heterointerface in the electrocatalysts [7, 31]. Song et al. fabricated Ni_3_N/Ni catalyst by optimizing the nitridation temperature and duration time of the electrodeposited Ni on Ni foam [32]. Increasing the duration time, the coverage of Ni_3_N increased, and the optimized nitridation resulted in the highest HER activity. As shown in Figure 3E, the heterointerface between Ni and Ni_3_N and the uniform distribution of the nitride phase is revealed in the TEM image and EDX mapping. These heterointerfaces promote the initial water adsorption, reducing the energy barrier for water dissociation [33]. The ammonolysis is also utilized to fabricate the nanostructured metal nitrides [34]. Li et al. introduced the electrospun Ti(OBu)_4_/PVP composite nanofibers as the precursor of TiN nanofibers, extending to VN, NbN, and ternary metal nitride nanofibers [35]. Also, Cheng et al. fabricated mesoporous oxides by impregnating the metal precursor into mesoporous silica, and the mesopores were maintained after rapid ammonolysis [36]. Furthermore, Jiang et al. fabricated 3‐dimensional (3D) ordered mesoporous CoN by applying the ordered mesoporous silica (KIT‐6) as a template for the oxide precursor [37]. Lai et al. synthesized the NiCo‐layered double hydroxide (LDH) nanoboxes by reacting Co‐based zeolitic imidazolate framework (ZIF‐67) nanocubes with Ni(NO_3_)_2_ under sonication [38]. These hollow nanoboxes of NiCo LDH precursor are observed in Figure 3F. After nitridation at a low temperature with ammonia, the NiCoN/C nanocages maintain the structure of the precursor without agglomeration. The enhanced specific surface area contributed to high mass activity for HER. Chen et al. similarly applied the nanostructures of ZIF‐67 to the OER catalyst as well as the HER catalyst after ammonolysis [39]. In addition to the advantages of nanostructures, the nitrogen vacancies formed during the nitriding process provided many active sites, improving the catalytic performances of both HER and OER.

### 3.2 Indirect Ammonolysis

**FIGURE 4:**
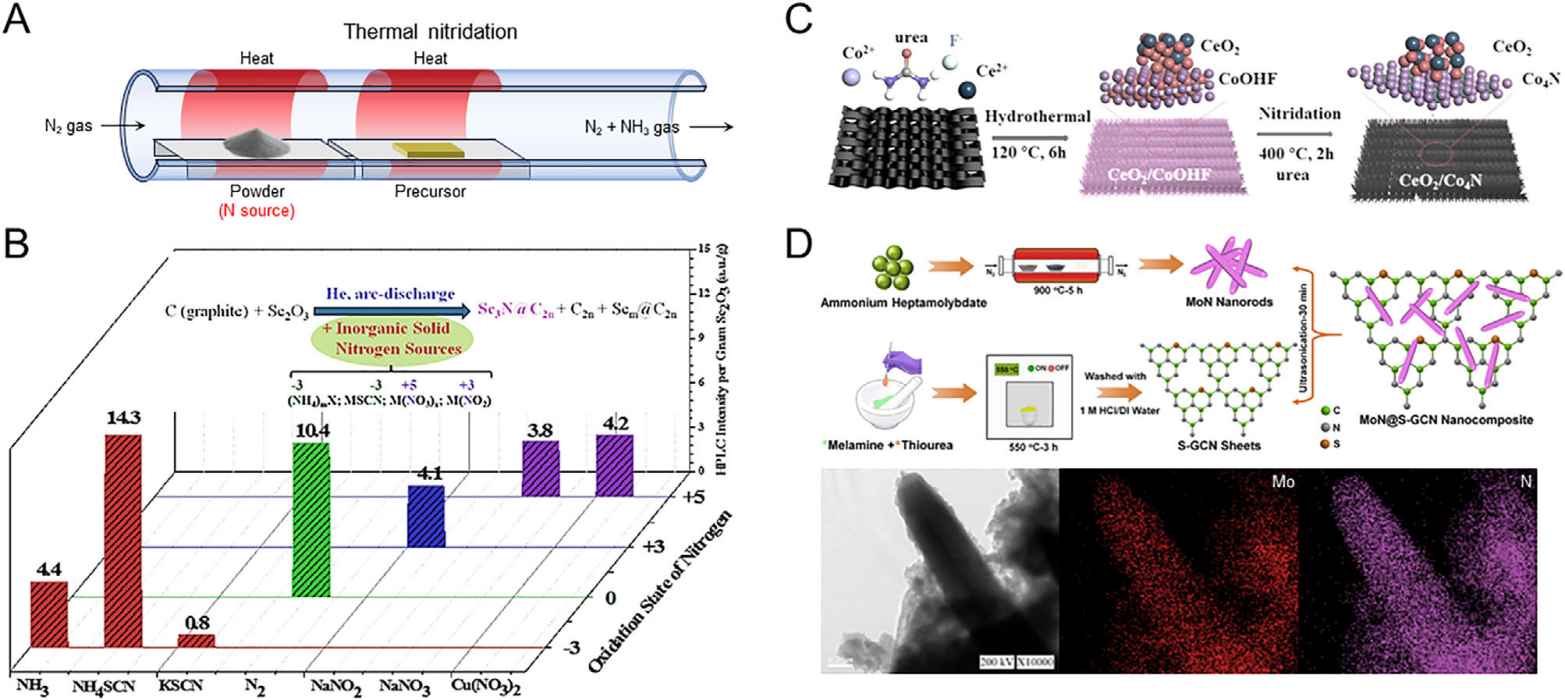
(A) Schematic illustration of the indirect ammonolysis. (B) Comparison of the HPLC intensity of fraction A per gram Sc_2_O_3_ obtained from different nitrogen sources. Reprinted with permission [41]. Copyright 2013, American Chemical Society. (C) The synthetic methods of CeO_2_/Co_4_N. Reprinted with permission [42]. Copyright 2020, Elsevier. (D) The synthetic process, HRTEM image and EDX mappings of MoN@S‐GCN nanocomposites. Reprinted with permission [46]. Copyright 2020, American Chemical Society.

Unlike direct ammonolysis using ammonia gas, the solid‐state powder is employed for indirect ammonolysis. Common nitrogen sources include urea, melamine, and other nitrogen‐rich compounds, which decompose upon heating and generate ammonia gas that indirectly nitrides the metal compound precursor [40]. Compared to gaseous ammonia in direct ammonolysis, the powder is easy to handle and inexpensive, making it advantageous for mass processing. However, controlling the phase and purity of the final product can be challenging, especially if the intermediate compound is not fully converted to nitride. Incomplete conversion can result in the presence of residual oxides or other phases. The synthetic process is illustrated in Figure 4A. When the powder is calcinated at a high temperature in an inert atmosphere, the nitrogen source decomposes to release ammonia or other nitrogen species, which then react with the metal compound precursor to form metal nitrides during heating. In order to suppress the formation of by‐products and obtain high purity and crystallinity of metal nitride, it is important to select an appropriate nitrogen compound. Liu et al. compared 12 nitrogen‐containing inorganic solid compounds with variable oxidation states of nitrogen and counter ions as nitrogen sources to synthesize Sc‐based metal nitride cluster‐fullerenes (Sc‐NCFs) [41]. As shown in Figure 4B, the yield of Sc‐NCFs per gram Sc_2_O_3_ precursor by using gaseous N_2_ and NH_4_SCN is much higher than those by using the nitrogen‐based group sources (thiocyanate, nitrates, and nitrites). The yield of Sc‐NCFs is highly dependent on the oxidation state of nitrogen. For the ammonia‐based group with (−3) oxidation states of nitrogen, the yield sensitively depends on the cation, unlike the nitrogen‐based group with (+3 or +5) oxidation states of nitrogen.

Yao et al. synthesized CeO_2_ nanoparticles on Co_4_N nanoarrays using urea as a nitrogen source [42]. As shown in Figure 4C, the CeO_2_/CoOHF nanorods precursor was obtained by hydrothermal synthesis. During the process, the hydrolyzed urea led to the formation of the nanorod‐like shape of the precursor, and the active nitrogen atoms decomposed from the urea indirectly nitrided the precursor to CeO_2_/Co_4_N at 450°C. Zhao et al. developed a facile route for metal nitride synthesis, extending a solid‐state metathesis method to various nitrides such as GaN, BN, CrN, VN, NbN, TaN, TaON, AlN, and TiN [43]. The Ga_2_O_3_ precursor was mixed with excess melamine, and the pellet was heated to 650°C in a low‐pressure environment and nitrided into GaN. Both hydrogen and carbon were involved in the reduction, making the nitridation process thermodynamically favorable. Similarly, Wang et al. fabricated nitrogen‐doped TiO_2_/Ti(C_0.7_N_0.3_)/nitrogen‐doped carbon photoelectrode. The solvothermally obtained TiO_2_ nanowires and melamine were mixed as a precursor and nitrogen source, respectively. After calcination, the Ti(C_0.7_N_0.3_) enhanced the photocurrent density of the N‐doped TiO_2_ photoanode as a cocatalyst [44]. Also, Hirai et al. synthesized four anti‐perovskite‐type compounds, ZnNNi_3_ and SnNCo_3,_ through nitridation reactions between metal oxides and melamine [45]. The decomposition of melamine generated radicals of carbon nitride, which combined with oxide precursors at high temperatures in evacuated quartz. In contrast, Jaysiva et al. adopted a method of reacting melamine and oxide precursors by the separated heat treatment [46]. As shown in Figure 4D, NH_4_Cl was placed in front of the (NH_4_)_6_Mo_7_O_24_ precursor in N_2_ flow. When the tube furnace was heated, NH_4_Cl was decomposed into HCl and ammonia, which indirectly nitrided (NH_4_)_6_Mo_7_O_24_ into the pure MoN. After ammonolysis, the MoN nanorods formed the nanocomposite with sulfur‐doped graphitic carbon nitrides. The EDX mapping revealed a uniform elemental distribution of MoN. In addition to melamine, nitrogen‐contained organic compounds are also used in indirect ammonolysis. Yang et al. utilized vanadium‐organic compounds (VAORCs) through solvent extraction using NaVO_3_ and dodecylamine [47]. After mixing with melamine, the VAORCs precursor was applied to two‐step heat treatment under the N_2_ atmosphere, transforming into nanocomposites of VN quantum dots and nitrogen‐doped hierarchical carbon. The use of melamine and dodecylamine as a mixed nitrogen source enabled sufficient reaction and uniform distribution of nitrogen atoms.

### 3.3 Solution Process

**FIGURE 5:**
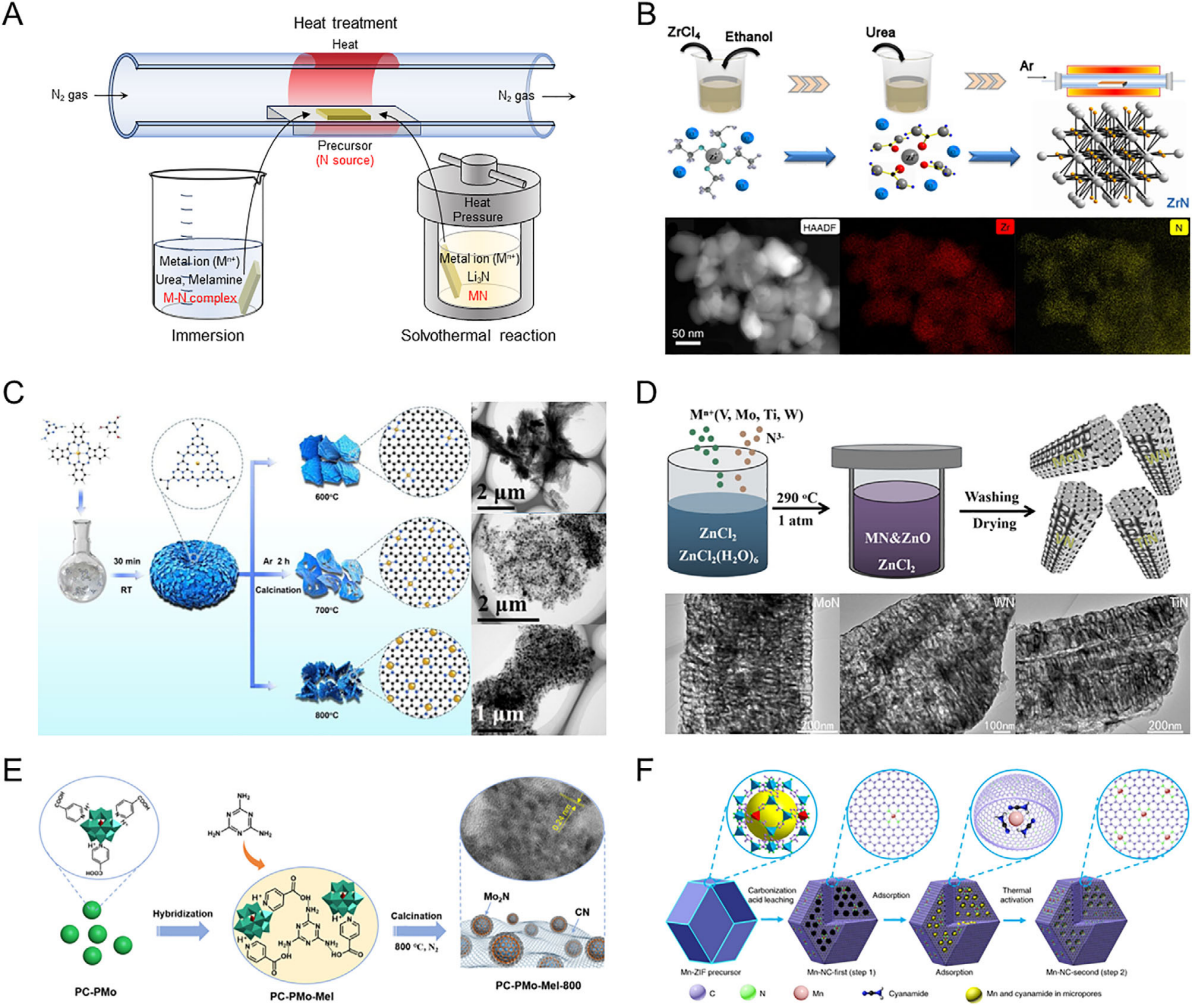
(A) Schematic illustration of the solution process and heat treatment. (B) The synthetic process, HAADF scanning TEM image, and EDX mapping ZrN nanoparticles. Reprinted with permission [51]. Copyright 2020, Nature Publishing Group. (C) The preparation process and TEM images of the series of CoO*_x_*/CoN*_y_* @CN*_z_* hybrids. Reprinted with permission [55]. Copyright 2020, Elsevier. (D) The synthesis and HAADF scanning TEM images of TMNs. Reprinted with permission [64]. Copyright 2021, Nature Publishing Group. (E) The preparation of the PC‐PMo‐Mel‐800 through two steps: hybridization and calcination. Reprinted with permission [70]. Copyright 2018, Wiley‐VCH GmbH. (F) The synthetic process of atomically dispersed MnN_4_ site catalyst. Reprinted with permission [71]. Copyright 2018, Nature Publishing Group.

The solution process is a versatile and widely used method for synthesizing metal nitrides. Solution processes occur at lower temperatures compared to ammonolysis, making them cost‐effective. Also, large quantities of metal nitrides can be produced by increasing the volume of the solution and maintaining reaction conditions. The solution process can yield metal nitrides with controlled particle sizes and morphologies, such as nanoparticles, nanorods, or thin films. However, since metal nitrides obtained from solution processes may have lower crystallinity compared to ammonolysis, additional heat treatment may be required to improve crystallinity. The metal precursor is supplied in the form of a cation in solution, where nitrogen‐containing compounds such as urea and melamine are included as nitrogen sources. The process of the solution‐based nitridation is schematically illustrated in Figure 5A. In the case of immersion, the metal‐nitrogen complex is constructed on the substrate in a solution containing metal ions and nitrogen sources. In the solvothermal process, which carries out the reactions at high temperatures and pressure for higher purity and crystallinity, metal salt and Li_3_N dissolved in an organic solvent are used as metal and nitrogen sources, respectively. The following heat treatment of the precursor in the form of powder or film in an inert gas forms M−N bonding or elevates crystallinity. Also, an additional nitriding process can be performed in an ammonia atmosphere.

In order to synthesize the Mo_2_N and W_2_N, Giordano et al. adopt a urea glass route, which is based on the gel‐like metal‐urea complex precursors. The method is not necessary for further purification and recrystallization processes [48]. Nakamura et al. also utilized the urea with copper acetate solution to synthesize Cu_3_N nanoparticles through composition optimization [49]. Ma et al. applied the urea‐glass route‐derived Mo_2_N to the HER catalyst [50]. Similarly, for the ORR catalyst, Yuan et al. synthesized ZrN nanoparticles with the urea‐glass route using ZrCl_4_ and urea as the metal and nitrogen sources, respectively [51]. As shown in Figure 5B, the complexation of Zr and urea was heated at 800°C under Ar flow, and the reductive urea was decomposed into NH_2_, NH_3_, HNCO, and H_2_NCO groups to form the ZrN phase. The EDX mapping image confirms the uniform nitridation of the ZrN nanoparticles. The urea‐glass route is also facile to synthesize ternary or higher‐order nitrides. Park et al. fabricated the Ni_2_Mo_3_N electrocatalyst by transferring the solution dissolving MoCl_5_ and urea with Ni foam [52]. The one‐step nitriding process is more economical compared with the two‐step annealing process, such as the solvothermal and nitridation methods. Beyond this, Jin et al. fabricated the high‐entropy metal nitride through the ball milling and urea‐glass route method [53]. The urea formed complexes with five metal salts of (V, Cr, Nb, Mo, Zr)Cl*_x_*, and the heat treatment transformed the metal‐urea glass into high entropy metal nitrides.

Melamine was also thermally decomposed into NH_3_, HCN, and H_3_NCN. Ma et al. introduced a melamine sponge as a substrate for an electrocatalyst since its highly porous structure is advantageous for absorbing the solution dissolving urea, glucose, and Fe(NO_3_)_3_ [54]. After heat treatment at 700°C under the Ar atmosphere, core‐shell Fe_3_N@C nanoparticles were conformally coated on 3D nitrogen‐doped carbon foam, which was derived from the strong coordination ability and adsorption capability of the melamine. The coordination ability of the melamine can be further enhanced by being used with another organic compound containing nitrogen atoms. Liu et al. first introduced a melamine‐cyanuric acid (MCA) complex as a nitrogen source, applying the strong π–π interaction between the MCA and cobalt phthalocyanine (CoPc) [55]. The overall preparation is presented in Figure 5C. The CoPc‐MCA complex was calcined at 600, 700, and 800°C under the Ar atmosphere and transformed into CoO*_x_*/CoN*_y_* @CN*_z_* hybrids. As shown in scanning electron microscopy (SEM) images, the size of the Co‐based nanoparticles in the nitride matrix increased as the temperature increased. The hybrids calcined at 700°C exhibited the most effective multifunctional electrocatalytic performances of HER, OER, and ORR in alkaline solution. These enhancements originated the large specific area and coordinate ability of the MCA network, which could not only anchor a number of CoPc nanoparticles but also prevent the catalytic site from leaching from the matrix. Furthermore, after heat treatment, CoO*_x_*/CoN*_y_* @CN*_z_* offered a synergistic effect from the multiple active sites, which benefits various catalytic reactions.

The solvothermal route is an effective technique for synthesizing metal nitrides at a lower temperature than ammonia‐based thermal nitridation [56]. The representative approach is solid‐state metathesis (SSM) using reactive solid nitrogen sources such as Li_3_N, LiNH_2_, NaN_3_, Ca_3_N_2_, and Mg_3_N_2_ [57]. The exothermic nature of the SSM reaction is advantageous for producing high‐quality metal nitrides [58]. The most commonly used nitrogen source is Li_3_N, and the general reaction can be represented as:

$$\mathrm{MCl}+Li_{3}N\to MN_{x}+\mathrm{LiCl}$$

where MCl and MN*_x_* are metal chloride and nitride. Xie et al. obtained nanocrystalline (30 nm) GaN at the highly low temperature of 280°C through the SSM reaction using Li_3_N and GaCl_3_ in benzene [59]. Similarly, Singh et al. used nickel bis(acetylacetonate) and Li_3_N as metal and nitrogen sources, respectively, to synthesize Ni_3_N nanosheets at 270°C under an Ar atmosphere [60]. In addition, Mazumder et al. synthesized nanocrystalline Cu_3_N particles at 200°C using the Na_3_N source [61]. By extension, the molten salt method, which utilizes molten salts as the reaction medium, is advantageous for achieving high‐purity nitrides and uniform particle sizes. Jin et al. synthesized 2‐dimensional (2D) layered metal nitrides by mixing metal oxide powders with alkali metal salts via ball milling [62]. After the precursor was melted under 5% ammonia in the Ar atmosphere, 2D layered metal nitrides were vertically grown on the surface of the molten mixture. The lower formation energy of the ion‐induced growth was facile to form 2D nitrides with high purity and crystallinity, and the optimized Mo_0.7_W_0.3_N electrocatalyst showed excellent HER performances. Guan et al. also synthesized 2D MoN nanosheets by the solvothermal and molten salt and solvothermal methods [63]. Various transition metal nitrides, such as MoN, WN, VN, and TiN nanosheets, were synthesized through the low‐temperature method, which reduces energy consumption. Furthermore, they extended the method to synthesize the 3D porous metal nitrides [64]. In order to reduce the nitridation barrier at a low temperature, chemically active Li_3_N was also used as a nitrogen source with (V, Mo, Ti, W)Cl*_x_* as the metal sources (Figure 5D). Also, ZnCl_2_ and ZnCl_2_·6H_2_O were introduced as the molten salt source because of their low melting point. After the solvothermal reaction at 290°C, the micron‐scale 3D porous VN, MoN, WN, and TiN were obtained. The ZnCl_2_ molten salt system reduced the melting temperature, and the ZnCl_2_·6H_2_O containing crystal water played the role of templates for the 3D porous metal nitrides. Hydrazine is also introduced to the solvothermal reaction as a nitrogen source because of its ability to release reactive nitrogen species, which contribute to the low process temperature. Zhu et al. synthesized Ni/Ni_3_C/Ni_3_N nanocomposites using hydrazine as a nitrogen source [65]. The composition of Ni_3_N and Ni_3_C was controlled by the hydrazine concentration.

In addition, various organic molecules are used as a nitrogen source in the solution process to synthesize metal nitrides. A representative example is an organic precursor containing amine groups. Vaughn et al. synthesized antiperovskite‐type colloidal Cu_3_PdN nanocrystals using oleylamine as the nitrogen source [66]. The precursor solution was prepared by mixing Cu(NO_3_)_2_·3H_2_O and Pd(acac)_2_ with oleylamine and 1‐octadecene, followed by the heat treatment at 240°C under the Ar atmosphere. The obtained Cu_3_PdN nanocrystals showed higher ORR activity than Cu_3_N and Pd. Similarly, Xi et al. used oleylamine with 1‐octadecylamine as the nitrogen source to synthesize the Cu_3_N ORR catalyst [67]. Yang et al. approached the metal nitride synthesis with a photochemical method [68]. The adenine having amine groups was used as nitrogen sources and dissolved with Co(NO_3_)_2_·6H_2_O precursor. When the solution was irradiated with a 125 W lamp for 24 h, the photosensitive adenine was decomposed into active fragments, which were combined with cobalt ions to form C/N/Co fragments. Without any thermal energy, the C/N/Co fragments self‐assembled, and Co_4_N nanoparticles were uniformly anchored on the surface of nitrogen‐rich carbons. Fechler et al. utilized an ionic liquid 1‐butyl‐3‐methylpyridinium dicyanamide (Bmp‐dca) as the nitrogen/carbon source [69]. The Bmp‐dca was mixed with TiCl_4_, VOCl_3_, or TiCl_4_/VOCl_3_ mixture and annealed at 240°C. The resulting TiN, VN, and TiVN nanoparticles with an average diameter of 5 nm were uniformly dispersed in the graphitic carbon matrix, increasing the surface area and active sites. Beyond the single ionic liquid, Li et al. introduced an ionic hybrid of organic‐polyoxometalate (POM), offering versatility in material synthesis [70]. The overall preparation process is presented in Figure 5E. The ionic pyridine‐4‐carboxylic acid (PC) was paired with a molybdenum‐based POM, PMo_12_O_40_^3−^ (PMo), with a 1:3 molar ratio. The PC‐PMo hybrid was additionally mixed with melamine in water and conjugated with amine groups in melamine. After the calcination at 800°C under the N_2_ atmosphere, the highly dispersed Mo_2_N nanoparticles on the carbon nitride substrate were acquired, showing remarkable catalytic ability for oxidative coupling of amines. Through the organic‐based synthesis, Li et al. successfully implemented atomic‐scale metal nitrides [71]. The synthetic process comprising two‐step doping and adsorption is illustrated in Figure 5F. In the first step, Mn‐doped ZIF‐8 precursors were carbonized and then leached with an acid solution to prepare a partially graphitized carbon host with optimal nitrogen doping and microporous structures. In the second step, additional Mn and N sources were adsorbed into the 3D carbon host, followed by thermal activation to generate an increased density of MnN_4_ active sites. The two‐step Mn doping‐adsorption method effectively increased the density of MnN_4_ active sites without the concomitant formation of Mn clusters, increasing the ORR activity.

### 3.4 Vapor Deposition

**FIGURE 6:**
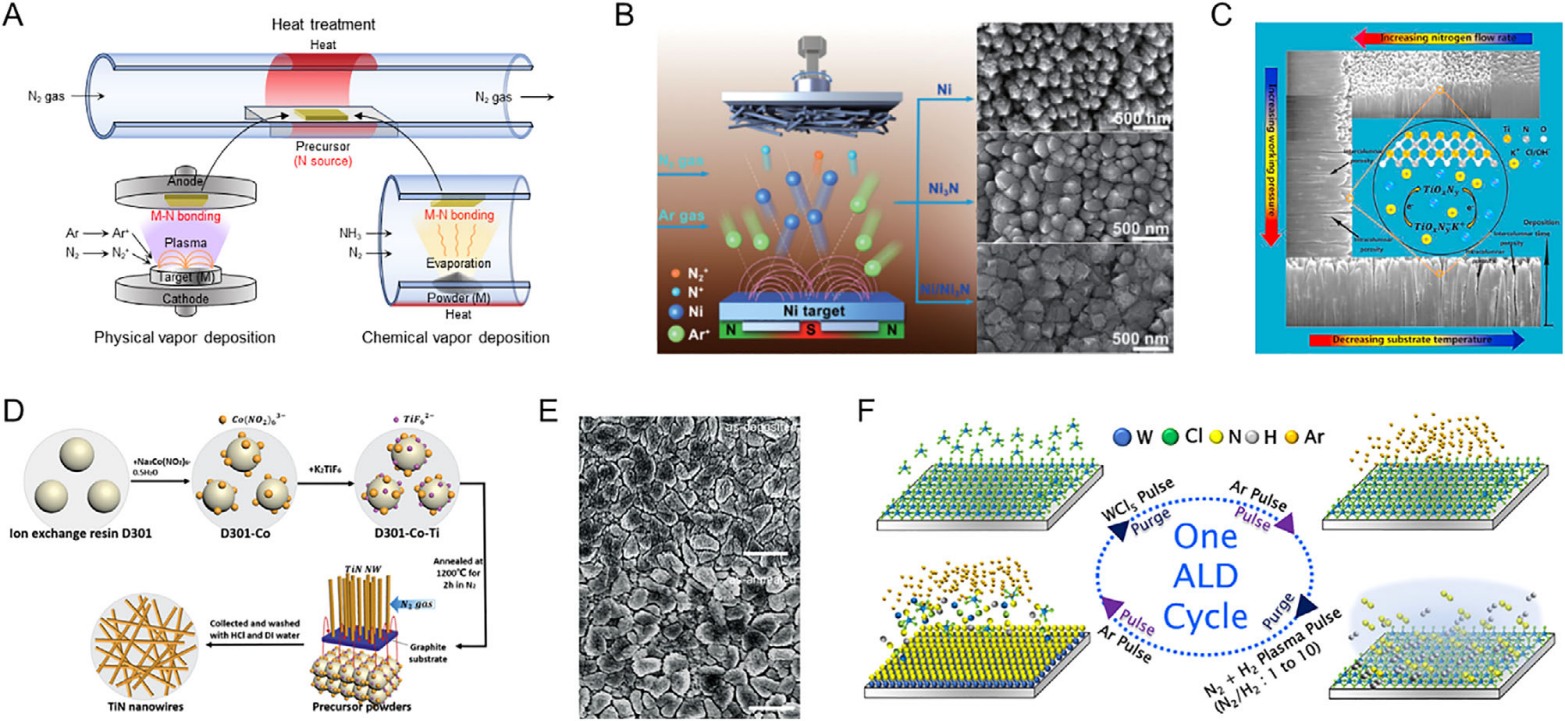
(A) Schematic illustration of the vapor deposition and heat treatment. (B) The synthesis and SEM images of Ni, Ni_3_N, and Ni/Ni_3_N thin films on CP through magnetron sputtering. Reprinted with permission [72]. Copyright 2023, Springer. (C) The surface chemistry and porosity of TiN thin films according to sputtering parameters. Reprinted with permission [76]. Copyright 2021, Elsevier. (D) The synthetic process of TiN nanowires. Reprinted with permission [79]. Copyright 2016, Royal Society of Chemistry. (E) SEM images of Ta_3_N_5_/Ta prepared by PCVD and Ta_3_N_5_/Ta after nitridation (Scale bar, 500 nm). Reprinted with permission [81]. Copyright 2020, Royal Society of Chemistry. (F) The overall PEALD WN*_x_* thin film deposition process and chemistry. Reprinted with permission [87]. Copyright 2023, American Chemical Society.

Vapor deposition is a widely used technique for synthesizing metal nitrides, offering precise control over the composition, thickness, and morphology of the resulting films. The process involves depositing metal and nitrogen species in a vapor phase onto a substrate, forming a nitride layer. However, the process parameters, such as temperature, pressure, and gas flow rates, need to be tightly controlled to achieve the desired film properties, increasing process complexity and decreasing cost‐effectiveness. As illustrated in Figure 6A, two main types of vapor deposition are used for metal nitride synthesis: physical vapor deposition (PVD) and chemical vapor deposition (CVD). The PVD is the physical transfer of material from a source to the substrate through evaporation or sputtering. In the case of sputtering, metal targets are prepared, and nitrogen gas is used as the nitrogen source. An inert gas (Ar) plasma bombards the metal target, expelling metal atoms into the vapor phases. Nitrogen gas is introduced into the chamber, where it reacts with the metal vapor to form metal nitrides on the substrate. The deposition parameters, such as gas flow rates, pressure, and power, are adjusted to control the film properties. The CVD is the chemical reaction of volatile precursors in the gas phase, which decompose and react on the surface of the substrate to form a metal nitride. Metal powder precursors, such as metal salt and organometallic compounds, are prepared, and nitrogen or ammonia gas flows as the nitrogen source for the target nitride phase. The precursors are heated in the reaction chamber and undergo a chemical reaction with nitrogen sources on the substrate, forming metal nitride. The properties of the nitride layer are precisely controlled by adjusting the reaction parameters, such as temperature, pressure, and gas flow rates. The deposited nitrides by PVD or CVD undergo additional heat treatment to enhance their crystallinity and conductivity.

The sputtering has excellence in deposition rate, versatility of deposition materials, and mechanical adhesion. In particular, transition metal nitrides for the electrocatalyst can be easily formed by sputtering. Qi et al. fabricated Ni/Ni_3_N heterostructure nanoarrays on carbon paper by magnetron sputtering [72]. As shown in Figure 6B, the Ni target was bombarded by Ar^+^ ions generated in plasma under magnetic fields, and the ejected atoms reacted with N^2+^ and N^+^ on carbon papers. According to N_2_/Ar flux ratio, the composition of the resulting films varied. The pure Ni films were obtained under a pure Ar atmosphere, while pure Ni_3_N films were deposited under a high N_2_/Ar flux ratio. Under a low flux ratio, the excess Ni atoms were deposited with Ni_3_N, causing the formation of Ni/Ni_3_N heterostructure. The SEM images represent that the N_2_/Ar flux ratio affected the morphologies as well as the composition of nitrides. The electron redistribution of the Ni/Ni_3_N heterostructure contributed to the electrocatalyst exhibiting remarkable HER activity. Furthermore, Murthy et al. introduced the doping strategy to metal nitride sputtering [73]. Mo_3_N_2_ and Ag, Cu, and V‐doped Mo_3_N_2_ films were fabricated using magnetron co‐sputtering. Ar and N_2_ gases were used as sputtering and reactive gases, and the power for Mo and dopant metals differed by 100 and 15 W, respectively. The uniform doping enhanced the charge transfer, and the Cu‐doped Mo_3_N_2_ catalyst showed the best HER activity. Titanium nitride is one of the most representative nitrides deposited by sputtering. Chang et al. constructed TiN thin films at room temperature by sputtering [74]. Also, Draher et al. introduced ion beam‐assisted sputtering to synthesize the TiN thin films [75]. Sun et al. precisely adjusted the morphology of the TiN film by controlling the sputtering parameters [76]. Figure 6C presents the modification of surface chemistry and porosity of TiN thin films according to the sputtering parameters. The nitrogen flow rate principally affected the bulk composition and surface chemistry. Also, the working pressure and substrate temperature changed the inter/intra‐columnar porosities of the films. The film thickness was controlled by the sputtering time. Tantalum nitride, a promising photoelectrode material, can also be fabricated by sputtering. Pihosh et al. performed nanostructuring using the sputtering method to improve the photoelectrochemical (PEC) characteristics of existing thin film‐based Ta_3_N_5_ [77]. The magnetron glancing angle deposition (GLAD) was utilized to fabricate the nanorod‐type 1‐dimensional (1D) TaO*_x_* precursors. The shadowing effect prevented the deposition of incident atoms behind previously formed islands, and the rotation contributed to forming nanorods perpendicular to the substrate. The rotation speed was optimized for more well‐separated and uniform nanorods, and the working gas pressure was lowered to prevent the collision of atoms and reduce the diameter of nanorods. In addition, Nandal et al. compared the morphology of Ta_3_N_5_ obtained under Ar and Ar/N_2_ plasma [78]. Although the Ta_3_N_5_ nanorods deposited under Ar plasma had a more aligned morphology than those under Ar/N_2_ plasma, the mixed plasma photoanode generated a higher photocurrent density. This represents that the difference in internal impurity formed according to gas flow during sputtering is more important than the surface morphology.

The CVD is advantageous in forming high‐purity metal nitride films and allows easy composition control. In particular, the CVD‐derived metal nitride could be formed in various morphologies from 1D to 3D. Han et al. directly fabricated 1D single‐crystalline TiN nanowires by the CVD method [79]. The synthetic process of TiN nanowires is presented in Figure 6D. The ion exchange resin D301 was added to a solution dissolving Na_3_Co(NO_2_)_6_·0.5H_2_O. The resin was adsorbed with Co(NO_2_)_6_^3−^ ions, and the resulting D301‐Co powder was mixed with the K_2_TiF_6_ precursor. The final D301‐Co‐Ti complexes were heated at 1200°C under the N_2_ atmosphere. From the resin powder exchanged with both Co(NO_2_)_6_^3−^ and TiF_6_^2−^ ions, TiN nanowires grew and exhibited good catalytic acidity for HER. Wang et al. synthesized ultrathin 2D WN crystals on SiO_2_/Si substrates via the CVD growth [80]. The mixed precursor of WO_3_ and NaCl was loaded into the chamber, where Ar/H_2_ and ammonia gases flowed at 800°C as the carrier gas and reaction gas, respectively. The concentration of ammonia not only tuned the thickness of WN crystals but also transformed the structure from 2D to 3D. The CVD approach is also used to synthesize the Ta_3_N_5_ for the photoelectrode. Nurlaela et al. demonstrated a new CVD route to form the Ta_3_N_5_ thin films, as shown in Figure 6E [81]. Ta_3_N_5_ was deposited on the Ta substrate through a plasma‐enhanced chemical vapor deposition. As the metal source, tertiary‐butylimido‐tris‐(ethylmethylamino) tantalum was heated in the chamber, and a radio frequency plasma was generated with N_2_ gas flow. Since the as‐deposited Ta_3_N_5_/Ta film was amorphous, the thermal treatment was carried out at 800°C under ammonia flow, converting it into a multilayer structure with high PEC efficiencies.

Atomic layer deposition (ALD) is a specialized technique within the realm of CVD that allows for the precise control of thin film deposition at the atomic level [82]. It is effective for synthesizing high‐quality metal nitrides due to its ability to uniform coatings with excellent film thickness and composition control. Unlike traditional CVD, which relies on continuous gas flow and reactions, ALD cyclically introduces precursor gases, ensuring that only a single atomic layer of material is deposited in each cycle [83]. The substrate is first exposed to a metal precursor gas, which chemisorbs onto the substrate surface, forming a monolayer. Then, an inert gas is introduced to purge any excess precursor and reaction by‐products from the chamber. The substrate is then exposed to a nitrogen precursor, ammonia gas, which reacts with the chemisorbed metal layer to form the metal nitride. Another purge step is performed to remove excess nitrogen precursor and by‐products, and these steps are repeated in cycles. Longrie et al. synthesized TiN thin films by using the thermal and plasma‐enhanced ALD (PEALD) using tetrakis(dimethylamino) titanium and ammonia as precursors [84]. Kim et al. also deposited TiN thin films using C_12_H_23_N_3_Ti and N_2_ plasma as Ti precursor and reactant gas, respectively [85]. The temperature window of the PEALD was lower than that of TiN films using TiCl_4_ precursor and ammonia gas. In addition, the ternary CoTiN alloy was fabricated by the supercycle method alternating Co and Ti precursor [86]. Seo et al. developed A PEALD process of WN*_x_* thin films using a fluorine‐free tungsten precursor WCl_5_ and N_2_/H_2_ mixture plasma [87]. As illustrated in Figure 6F, the ALD cycle consisted of the WCl_5_ pulse, N_2_/H_2_ plasma pulse, and in between Ar purging. The N_2_/H_2_ gas ratio was controlled to adjust the stoichiometry of the WN*_x_* thin film, and both the hexagonal WN phase and cubic W_2_N phase were obtained.

## 4 Catalysis via Metal Nitride Catalysts

### 4.1 Electrocatalysis

Metal nitrides exhibit versatility as catalytic materials for various electrochemical energy conversion reactions with their distinct structural diversity and associated electronic configurations. This section explores the forms and mechanisms of metal nitride‐based electrocatalysts to achieve catalytic activity in specific redox reactions. We will provide a detailed overview of their practical applications in the hydrogen evolution reaction (HER), oxygen evolution reaction (OER), oxygen reduction reaction (ORR), nitrogen reduction reaction (NRR), carbon dioxide reduction reaction (CRR), and biomass oxidation reaction (BOR). The metal nitride catalysts studied in each electrochemical conversion reaction and their performances are summarized in Table 1.

**TABLE 1:** Electrocatalysis performance of various metal nitride electrocatalysts under different reaction conditions.

Hydrogen evolution reaction (HER)					
		Overpotential) (mv)			
Catalysts	Electrolytes	*η*_10_	*η*_100_	Tafel slope (mV dec‐^1^)	Stability (h)
Ni_3_N nanoparticles [92]	1 M KOH	68	–	–	2.8
TiN nanowires [79]	1 M HClO_4_	≈180	–	54	100
[001] Ta_5_N_6_ single crystal [94]	0.5 M H_2_SO_4_	84	–	–	120
2D *h*‐W_2_N_3_ nanosheets [95]	0.5 M H_2_SO_4_	98.2	–	59	–
	0.5 M H_2_SO_4_	≈180	–	–	10
2D Mo_5_N_6_ nanosheets [20]	1 M KOH	94	–	66	10
	Natural seawater	258	–	–	100
Co_0.6_Mo_1.4_N_2_ [96]	0.1 M HClO_4_	200	–	–	–
Ni_0.2_Mo_0.8_N [97]	1 M KOH	83	–	70.7	12
V_0.2_Mo_0.8_N_1.2_ [98]	0.5 M H_2_SO_4_	158	–	39	100
P, W co‐doped Co_3_N [100]	1 M KOH	41	–	40	25
Ni_3_N/Pt [102]	1 M KOH	50	–	365	60
MoN‐5% Os [103]	0.5 M H_2_SO_4_	12.3	–	30	20
	1 M PBS	32.7	–	47.2	20
	1 M KOH	21.2	–	34.4	20
Ni_3_N/Ni/NF [32]	1 M KOH	12	64	–	24
Co/WN [105]	1 M KOH	27	117	77	50
Co_4_N‐CeO_2_ [106]	1 M KOH	24	–	61	50
Ni_3_N@2M‐MoS_2_ [108]	1 M KOH	–	97	43.2	300

Oxygen evolution reaction (OER)						
		Overpotential (mV)				
Catalysts	Electrolytes	*η*_10_	*η*_100_	*η*_500_	Tafel slope (mV dec^−1^)	Stability (h)
Co_4_N nanowires [113]	1 M KOH	257	–	–	44	12
Co_4_N [114]	1 M KOH	330	–	–	58	3.6
Co_4_N_0.82_ nanosheets [115]	1 M KOH	190	–	–	84	–
Ni_3_N nanosheets [117]	1 M KOH	256	–	–	45	18
Mn_3_N_2_ [118]	1 M KOH	270	390	–	101	24
Co_0.15_Fe_0.85_N_0.5_ [119]	1 M KOH	266	–	–	30	90
Ni_2_Co‐N nanocactoids [121]	1 M KOH	143	–	–	53	60
FeNi_3_‐N [123]	1 M KOH	222	307	–	41.53	36
CoVFeN [124]	1 M KOH	212	264	–	34.8	100
Co_3_Mo_3_N [125]	1 M KOH	290	–	–	60	24
Co_2.5_Fe_0.5_Mo_3_N [126]	1 M KOH	218	≈270	–	41	90
NiO/CoN nanowires [131]	1 M KOH	300	–	–	35	12
Co_4_N@N‐doped carbon [132]	1 M KOH	257	–	–	58	72
NiMoN/NiFe LDH [133]	1 M KOH	–	–	236	42.2	200
IrO*_x_*/TiN (60 µg_Ir_ cm^−2^) [134]	0.1 M HClO_4_	293	–	–	40.8	200

Oxygen reduction reaction (ORR)					
		*E*_1/2_	*J*_L_	Tafel slope	Stability
Catalysts	Electrolytes	(V vs. RHE)	(mA cm^−2^)	(mV dec^−1^)	(h)
3DOM Co@WNO [141]	0.1 M KOH	0.85	≈5.9	48	160
Cu_3_PdN nanoparticles [66]	0.1 M KOH	≈0.93	–	–	10,000 cls
Fe*_x_* N/N‐doped carbon nanotube–graphene [144]	0.1 M KOH	0.89	≈5.5	58	10000 cls
Co_4_N@NC [145]	0.1 M KOH	0.824	≈5.5	64.92	750
(Co_0.17_Fe_0.83_)_3_N@N‐doped porous carbon [149]	0.1 M KOH	0.882	6.267	64.2	160
Co_3_N/C [150]	1 M KOH	0.862	3.7	37	10,000 cls
Co_4_N/C [151]	1 M KOH	0.875	3.8	49	10,000 cls
ZrN [51]	0.1 M KOH	0.80	5.18	–	100

Nitrogen reduction reaction (NRR)					
			FE	*V*	Stability
Catalysts	Electrolytes	NH_3_ yield	(%)	(V vs. RHE)	(h)
VN [156]	0.05 M H_2_SO_4_	3.3 × 10^−10^ mol s^−1^ cm^−2^	6.0	−0.1	116
MoN nanosheets [158]	0.1 M HCl	3.01 × 10^−10^ mol s^−1^ cm^−2^	1.15	−0.3	27
Mo‐vacancy‐rich MoN@					
N‐doped porous carbon	0.1 M HCl	76.9 µg h^−1^ ${\mathrm{mg}}_{\mathrm{cat}.}^{-1}$	6.9	−0.2	48
(MV‐MoN@NC) [159]					
Mo_2_N‐BN [161]	0.1 M H_2_SO_4_	33.7 µg h^−1^ ${\mathrm{mg}}_{\mathrm{cat}.}^{-1}$	61.5	−0.3	20
nitrogen vacancies on W_2_N_3_					
(NV‐W_2_N_3_) [162]	0.1 M KOH	11.66 µg h^−1^ ${\mathrm{mg}}_{\mathrm{cat}.}^{-1}$	11.67	−0.2	24
N‐doped carbon layer (NC)/	0.1 M Na_2_SO_4_	76.15 µg h^−1^ ${\mathrm{mg}}_{\mathrm{cat}.}^{-1}$(−0.8 V)	24.60	−0.5	10
Bi single atoms (SAs) /TiN [163]					

Carbon dioxide reduction reaction (CRR)					
		*J*	FE	*V*	Stability
Catalysts	Electrolytes	(mA cm^−2^)	(%)	(V vs. RHE)	(h)
Pd/NbN [164]	0.5 M NaHCO_3_	CO: −0.05	CO: 38	−0.6	3
Cu–Ni/TiN [165]	0.1 M NaHCO_3_	−9.0	CO: 19.1	−0.89	24
			HCOO^–^: 6.7		
Cu_3_N [166]	0.1 M KHCO_3_	−30	C_2_H_4_: 60	−1.6	20
Cu/Cu_3_N [167]	0.1 M KHCO_3_	C_2+_: −14	C_2+_: 64	−0.95	30
Cu/Cu_3_N [168]	0.1 M CsHCO_3_	C_2+_: −18.5	C_2+_: 68	−1.0	3.3
In_2_O_3_/Cu_3_N [169]	0.1 M KHCO_3_	CO: −3.0	CO: 65	−0.6	50
InN–C [170]	0.5 M KHCO_3_	CO: −8.4	CO: 92.2	−0.8	–

Biomass oxidation reaction (BOR)					
		*V*	FE	Tafel slope	Stability
Catalysts	Electrolyte	(V vs. RHE)	(%)	(mV dec^−1^)	(h)
Ni‐Mo‐N [172]	1 M KOH	1.36 @ 10 mA cm^−2^	Formate, 95.0	87	12
	+ 0.1 M glycerol				
Mn‐CoN [173]	1 M KOH	1.19 @ 10 mA cm^−2^	Formate, 97.7	89	70
	+ 0.3 M glycerol				
CuCoN_0.6_ [174]	1 M KOH	1.07 @ 10 mA cm^−2^	Formate, 90.0	179.3	60
	+ 0.1 M glycerol				
Ni_3_N/Co_3_N nanowires [31]	1 M KOH	1.30 @ 20 mA cm^−2^	Formate, 94.6	131.4	200
	+ 0.1 M glycerol				
VN [176]	1 M KOH	1.36 @ 10 mA cm^−2^	FDCA, 84	–	3
	+ 10 mM HMF				
Ni_3_N@C [177]	1 M KOH	1.38 @ 50 mA cm^−2^	FDCA, 98	48.9	15
	+ 10 mM HMF				

*Note*: η_10,_ *η*_100_, and *η*_500_ refer to the overpotential at the current density of 10, 100, and 500 mA cm^−2^, respectively; ***E***_1/2_ refers to the half‐wave potential for the ORR; *J*_L_ refers to the limiting‐current density for the ORR.

#### 4.1.1 Hydrogen Evolution Reaction

Hydrogen evolution reaction (HER) involves three possible reaction steps: the Volmer step (electrochemical hydrogen adsorption), the Heyrovsky step (electrochemical desorption), and the Tafel step (chemical desorption). A series of HER, utilizing two electrons to produce a hydrogen molecule, occurs through either the Volmer–Tafel or Volmer–Heyrovsky reaction pathways [88]. Each elementary step appears as follows depending on the pH of the electrolyte:

$$\text{In acidic media}:M+H^{+}+e^{-}\to M-H^{*}\text{(Volmer step)}$$

$$M-H^{*}+M-H^{*}\to H_{2}+2M\;\left(\text{Tafel step}\right)$$

$$M-H^{*}+H^{+}+e^{-}\to H_{2}+M\;\text{(Heyrovsky step)}$$

$$\text{In alkaline media}:\;M+H_{2}O+e^{-}\to M-H^{*}+{\mathrm{OH}}^{-}\;\text{(Volmer step)}$$

$$M-H^{*}+M-H^{*}\to H_{2}+2M\;\text{(Tafel step)}$$

$$M-H^{*}+H_{2}O+e^{-}\to H_{2}+M+{\mathrm{OH}}^{-}\;\text{(Heyrovsky step)}$$

The HER catalyst exhibits high activity when the adsorption of intermediates H^*^ is neither too strong nor too weak, in accordance with the Sabatier principle [89]. Therefore, an ideal HER catalyst has a near‐zero Gibbs free energy for atomic hydrogen adsorption (Δ*G*_H*_) [90, 91]. The superior catalytic activity of Pt, a representative noble metal HER catalyst, is attributed to its optimum hydrogen binding energy. Indeed, noble metal catalysts have limitations in scalability, prompting significant research into alternate materials. Among them, metal nitrides with advantageous electronic structures provide opportunities for improving HER performance based on their structural diversity.

Basically, the electronic structure of metal nitrides, modified from their parent metals, directly changes the catalytic HER activity. Therefore, optimizing HER catalytic performance necessitates understanding how N sites affect adsorption energy, electrical conductivity, and active sites. The introduction of N primarily alters the metal's valence state, leading to changes in hydrogen adsorption energy. For example, Ni, which has relatively strong hydrogen adsorption, can weaken this adsorption and enhance HER activity through nitridation to form the Ni_3_N phase. According to DFT calculations by Wang et al., Bader charge analysis showed that the formation of interstitial nitrogen oxidizes nickel, increasing its partial positive charge from +0.22 to +0.6 [92]. Consequently, Ni_3_N has a lower Δ*G*_H*_ value than Ni, bringing it closer to the Δ*G*_H*_ value of Pt. This trend also results in a decreased barrier height in the water dissociation pathway, demonstrating its utility in alkaline HER as well [93]. Additionally, the excess *d*‐electrons of metals give metal nitrides high conductivity. Low charge transport resistance has been shown to improve HER performance in phases such as TiN [79] and Ta_5_N_6_ [94] phases. This enhancement can be further strengthened by achieving high crystallinity, controlling morphology, and exposed facets.

**FIGURE 7:**
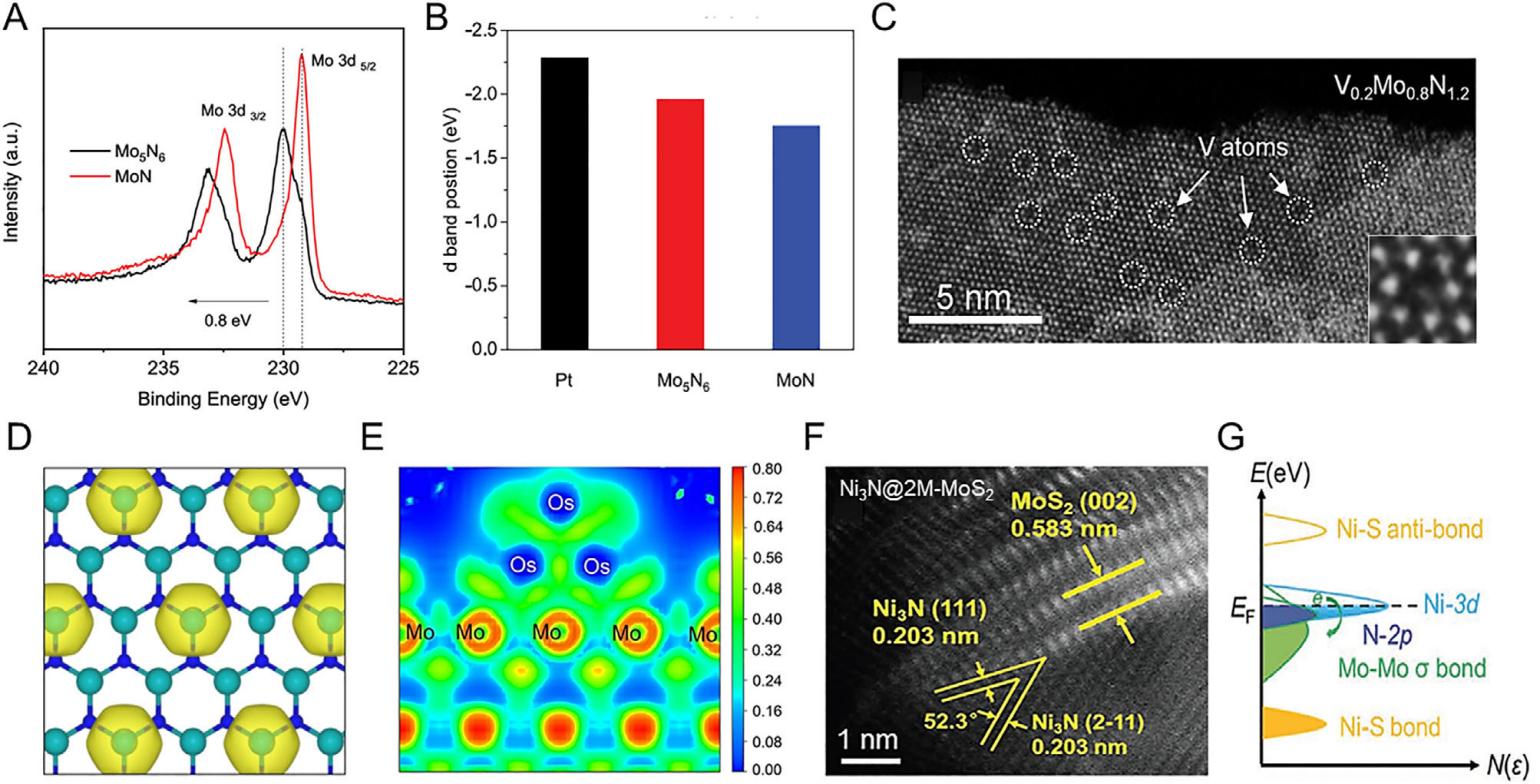
(A) Mo 3d XPS spectra of MoN and Mo_5_N_6_. (B) d band center position of Pt (111), Mo_5_N_6_ and MoN. Reprinted with permission [20]. Copyright 2018, American Chemical Society. (C) HAADF‐STEM image of 2D V_0.2_Mo_0.8_N_1.2_ solid solution. The white‐color dot‐circles highlight V atoms and poor contrast compared with Mo atoms. (D) Differential charge density of V‐doped MoN_1.2_. The yellow contour represents electron accumulation. The iso‐surface level is set to be 0.05 e Bohr^−3^. Reprinted with permission [98]. Copyright 2022, Wiley‐VCH GmbH. (E) Electron localization function of MoN‐Os. Reprinted with permission [103]. Copyright 2023, Elsevier. (F) HAADF‐STEM image of Ni_3_N@2M‐MoS_2_. (G) Proposed charge regulation effect on manipulating active electronic states in Ni_3_N@2M‐MoS_2_. Reprinted with permission [108]. Copyright 2022, Wiley‐VCH GmbH.

Most metal nitrides have thermodynamically favorable *N*‐deficient phases with an *N*/*M* ratio of less than 1. However, for some metals, exceptional HER activity and durability have been reported through the successful synthesis of *N*‐rich phases. The synthesis of ultrathin 2D *h*‐W_2_N_3_ nanosheets on KCl surfaces using domain‐matching epitaxy is facilitated by reduced interfacial formation energy, as revealed by theoretical calculations. These nanosheets with abundant W‐N bonding exhibit enhanced catalytic HER activity compared to their bulk counterpart [95]. In Mo‐based nitrides, the Ni‐catalyzed salt‐templated method enabled the successful synthesis of atomically thin 2D nanosheets of Mo_5_N_6_. The increased nitrogen content within the synthesized Mo_5_N_6_ lattice resulted in a higher valence state of Mo compared to MoN through electronic redistribution (Figure 7A). Also, the calculated *d*‐band center of Mo_5_N_6_ was −1.96 eV, closer to Pt's −2.28 eV than MoN's −1.75 eV (Figure 7B). This advantageous electronic structure of Mo_5_N_6_ demonstrated activity and stability in various electrolytes and seawater, operating stably for 100 h at an overpotential of 300 mV in natural seawater. This demonstrates that the high valence state of the N‐rich phase increases corrosion resistance, compensating for the oxidation vulnerability of the N‐deficient phase [20].

The strategy of enhancing HER activity by incorporating two or more metals into nitrides has also been actively researched. Specifically, efforts have been made to improve HER activity through compositional adjustments in Mo‐based nitrides, such as Co_0.6_Mo_1.4_N_2_ [96] and Ni_0.2_Mo_0.8_N [97]. For nitrogen‐rich phases, the bimetallic composition of Mo_0.7_W_0.3_N_1.2_ [62] and V_0.2_Mo_0.8_N_1.2_ [98] enhanced HER catalytic properties compared to MoN_1.2_. The V_0.2_Mo_0.8_N_1.2_ synthesized via the molten‐salt method exhibited the same hexagonal symmetry of the planes as MoN_1.2_. In the STEM image, V atoms are distributed within a single layer, showing a distinct contrast with Mo (Figure 7C). The V_0.2_Mo_0.8_N_1.2_ showed an overpotential of 158 mV at 10 mA cm^−2^ in acidic electrolytes and stability over 100 h. Furthermore, V_0.2_Mo_0.8_N_1.2_ exhibited a Tafel slope of 39 mV dec^−1^, significantly lower than 97 mV dec^−1^ of MoN_1.2_, confirming its favorable shift toward an electronic structure conducive to HER. The alloying of V promoted electron transfer from V to Mo, thereby accelerating the Volmer step at electron‐rich active sites on the catalyst surface (Figure 7D) [98]. Even lower doping levels with heterogeneous metal elements induce changes in the electronic structure that facilitate hydrogen and water adsorption [99]. In the PW‐Co_3_N electrocatalyst, where P substitutes for N and W substitutes for Co, the *d*‐band center shifted down to −1.54 eV compared to −1.41 eV in pure Co_3_N. The co‐doping effect not only reduced the hydrogen absorption energy but also enhanced H_2_O adsorption. Consequently, PW‐Co_3_N exhibited a low overpotential of 41 mV at 10 mA cm^−2^ in alkaline electrolytes [100].

Metal nitride heterostructures offer abundant active sites at heterogeneous interfaces. A representative approach is to use a nitride framework decorated with Pt nanoparticles, which utilizes Pt's high HER activity while reducing the Pt loading amount [101]. Wang et al. developed Pt‐decorated Ni_3_N nanosheets with a low noble metal content of 15% as an alkaline HER electrocatalyst [102]. A metallic Ni_3_N acted as an electron channel, Ni(OH)_2_ formed thinly on the surface as an H_2_O dissociation site, and Pt as an H^*^ adsorption site, forming a favorable overall reaction path. Similarly, the MoN‐Os catalyst, which uses Os as a cost‐effective alternative to Pt, outperformed Pt/C across a wide pH range. The electronic localization function (ELF) between Mo and Os demonstrates strong electronic interaction with Os nanoclusters and 2D‐MoN, proving its superiority in electron transport and deterioration prevention (Figure 7E) [103]. Hu et al. developed ultra‐low Pt metal nitride catalysts for seawater hydrogen production [104]. The hydrothermally synthesized NiMoN was utilized as a support for ultra‐low Pt (0.07 wt%)–Ni catalyst. The composition‐controlled Pt–Ni@NiMoN catalyst exhibited overpotentials of 11 and 90 mV to reach 10 and 500 mA cm^−2^ in seawater. In seawater and highly chlorinated NaCl, the Pt–Ni@NiMoN catalyst showed high durability for 200 h, which originated a strong anchoring effect between Pt and metal nitride.

Recent studies reveal that controlled nitridation of oxides or metal precursors forms heterogeneous active sites, optimizing water dissociation and hydrogen binding energies for alkaline HER. For example, the interfacial sites of Ni_3_N/Ni obtained by nitriding electrodeposited Ni in ammonia showed excellent alkaline HER properties, requiring only 12 mV of overpotential to achieve a current density of 10 mAcm^−2^ [32]. In addition, the Co/WN [105] heterostructure derived from CoWO polyhedral and the Co_4_N‐CeO_2_ [106] heterostructure derived from Co(OH)_2_‐CeO_2_ nanosheets showed effective alkaline HER activity. Also, heterojunction with sulfide has been reported [107]. For example, Ni_3_N@2M‐MoS_2_, a heterostructure of Ni_3_N and monoclinic 2M‐MoS_2_, showed a notably low overpotential of 155 mV to reach 1 A cm^−2^ in alkaline conditions (Figure 7F). When the two metallic phases are chemically bonded, the charge of Ni and N atoms moves as the Mo─Mo bond downshifts. Then, the electronic states of Ni 3*d* and N 2*p* around the Fermi level promote electron donation for HER (Figure 7G). According to the calculated energy barrier, the Ni site of Ni_3_N@2M‐MoS_2_ was an active site for water dissociation, whereas the N site was an active site for hydrogen adsorption. These unique electronic features overcome the shortcomings of single metal nitrides and ultimately enable stable operation for 300 h [108].

#### 4.1.2 Oxygen Evolution Reaction

Oxygen evolution reaction (OER), another half‐reaction of water splitting, is a kinetically sluggish reaction requiring more overpotential compared to HER. The complex multistep reaction involving four electrons is traditionally explained by the adsorbate evolution mechanism (AEM) and the lattice oxygen‐mediated mechanism (LOM). The AEM considers the metal atom as the active site and has the following reaction steps depending on pH [109].

$$\mathrm{In}\mathrm{acidic}\mathrm{media}:H_{2}O+M^{*}\to HO^{*}+H^{+}+e^{-}$$

$$HO^{*}\to O^{*}+H^{+}+e^{-}$$

$$O^{*}+H_{2}O\to \mathrm{HO}O^{*}+H^{+}+e^{-}$$

$$\mathrm{HO}O^{*}\to O_{2}+H^{+}+e^{-}+M^{*}$$

$$\text{In alkaline media}:OH^{-}+M^{*}\to HO^{*}+e^{-}$$

$$HO^{*}+OH^{-}\to O^{*}+H_{2}O+e^{-}$$

$$O^{*}+OH^{-}\to \mathrm{HO}O^{*}+e^{-}$$

$$\mathrm{HO}O^{*}+OH^{-}\to O_{2}+H_{2}O+e^{-}+M^{*}$$

OER activity in AEM is predominantly influenced by the binding energy difference between metal atoms and reaction intermediates. According to the linear correlation of the binding energies of the HO* and HOO* intermediates, the Δ*G*_O*_−Δ*G*_HO*_ value is used as a descriptor to predict OER activity [110]. On the other hand, LOM does not confine the active center to the metal center but extends it to two adjacent metal sites. The LOM pathway involves the formation of metal‐oxo species and their direct combination to produce O_2_ [111]. Until now, the development of active sites, mainly for oxides and hydroxides, has reduced the OER overpotential [112]. However, metal nitrides have great potential as OER catalysts because they can overcome the limitation of low electrical conductivity in conventional materials and provide abundant active sites due to their unique electronic structure.

**FIGURE 8:**
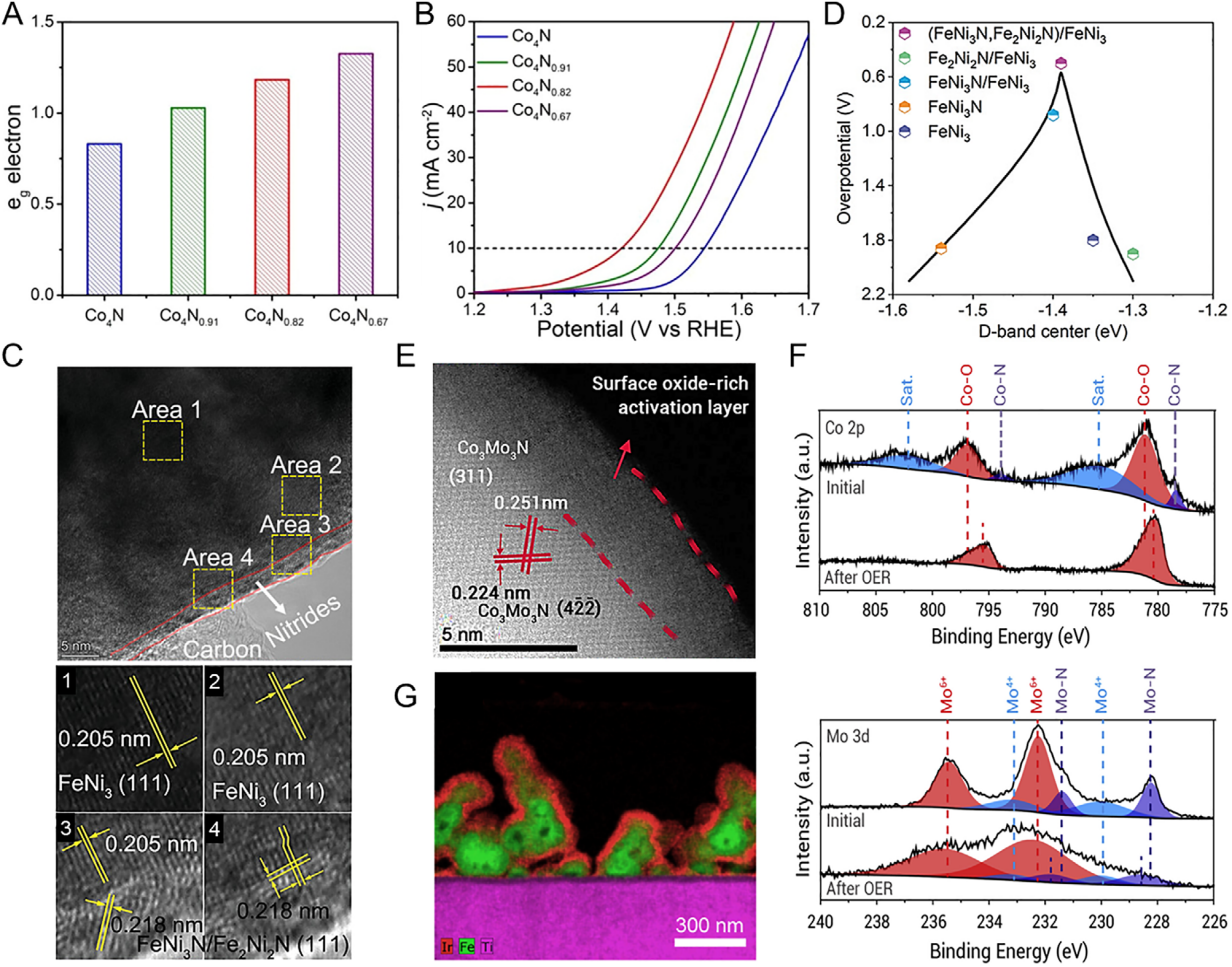
(A) The average number of *e*_g_ electrons of pristine Co_4_N, Co_4_N_0.91_, Co_4_N_0.82_, and Co_4_N_0.67_ nanosheets. (B) *J*–*V* curves of pristine Co_4_N, Co_4_N_0.91_, Co_4_N_0.82_, and Co_4_N_0.67_ nanosheets in 1 M KOH electrolyte. Reprinted with permission [115]. Copyright 2021, Elsevier. (C) HR‐TEM image of (FeNi_3_N, Fe_2_Ni_2_N)/FeNi_3_ and magnified images from yellow dashed box area 1–4. (D) Volcano profile of the calculated d‐band centers and theoretical overpotentials of Fe_2_Ni_2_N/FeNi_3_, FeNi_3_, (FeNi_3_N, Fe_2_Ni_2_N)/FeNi_3_, FeNi_3_N/FeNi_3_, and FeNi_3_N. Reprinted with permission [123]. Copyright 2022, Wiley‐VCH GmbH. (E) HR‐TEM image of Co_3_Mo_3_N after OER cycles. (F) Co 2p region and Mo 3d region XPS spectra of Co_3_Mo_3_N before and after OER cycles. Reprinted with permission [125]. Copyright 2021, Cell Press. (G) EDS image of the electrodeposited iridium‐oxide film on Fe_2_N‐coated titanium surface. Reprinted with permission [135]. Copyright 2023, Elsevier.

The initial investigation evaluated the OER activities of crystalline Co_3_O_4_ and Co_4_N synthesized through thermal oxidation and nitridation of Co(OH)F nanowires, respectively. The metallic nitride, which has higher electrical conductivity than the semiconducting oxide, was advantageous for charge transfer from the catalyst surface to the entire electrode. Co_4_N showed a reduced overpotential of 257 mV at a current density of 10 mAcm^−2^, compared to an overpotential of 320 mV for Co_3_O_4_ [113]. In Co‐based nitrides (Co_2_N, Co_3_N, and Co_4_N), unlike other common metal nitrides, it has been reported that electrical conductivity increases as the nitrogen content decreases. This finding indicates that changes in the overlap of electron clouds can also affect conductivity. As N deficiency increases, electron delocalization is strengthened, leading to improved conductivity. Therefore, the excellent intrinsic electrical conductivity of Co_4_N supports higher OER activity than Co_2_N and Co_3_N [114]. Subsequently, Liu et al. further reduced the nitrogen content in Co_4_N, confirming the OER activity trend according to nitrogen content [115]. The hydrogen atmosphere annealing time of pristine Co_4_N controlled the formation of nitrogen vacancies, resulting in Co_4_N_0.91_, Co_4_N_0.82_, and Co_4_N_0.67_ phases. As the nitrogen content of Co_4_N decreases, the number of *e*_g_ electrons increases (Figure 8A). Previously, it was revealed that active Co^3+^ sites have the electronic configuration of $t_{2g}^{6}e_{g}^{0}$ at a low spin state or $t_{2g}^{4}e_{g}^{2}$ at a high spin state, and the *e*_g_ occupancy value at the summit of the volcano plot of OER activity is 1.2 [116]. Based on this, the number of *e*_g_ electrons in Co_4_N_0.82_ is close to the optimal value, and in fact, it exhibited excellent OER activity with an overpotential of 190 mV at 10 mA cm^−2^ (Figure 8B). Similarly, in 2D Ni_3_N nanosheets, nitrogen vacancies generated by dimension reduction during thermal nitridation provide a larger surface area and more active sites than the bulk counterpart [117]. In addition, the Mn_3_N_2_ phase synthesized through ammonolysis of the molecular precursor showed an overpotential of 270 mV at 10 mA cm^−2^ and an operation stability of 180 h. TEM analysis after stability evaluation revealed the formation of a highly active MnO*_x_* shell on a conductive Mn_3_N_2_ core [118]. It is worth noting that, unlike HER, metal nitride‐based OER catalysts undergo surface reconstruction under oxidizing operating environments. Thus, the thermodynamic instability of metal nitrides in water oxidation is one of the important aspects to consider when designing metal nitride catalysts.

Based on well‐known OER active elements such as Co, Ni, and Fe, ternary and quaternary nitrides with controlled composition have also reported remarkable performance. An et al. investigated the OER activity of Co*_x_* Fe_1−_ *_x_* N_0.5_ by increasing the Co content in orthorhombic FeN_0.5_ to 20% [119]. OER activity was highest in Co_0.15_Fe_0.85_N_0.5_, and DFT calculations revealed that this composition maximizes the effect of the Co‐Fe site, which promotes the conversion of HO^*^ to O^*^ and O^*^−O^*^ coupling. Given that the fine proportions of the metal compositions directly influence the catalytic OER activity, composition optimization is obviously required [120]. Various compositions such as Ni‐Co, Ni‐Fe, and Ni‐Mn have also been evaluated for OER activity [121, 122]. For Ni‐Fe, a characteristic alloy composition for OER, nitriding FeNi_3_ alloy resulted in the synthesis of dual‐phase nitrides of FeNi_3_N and Fe_2_Ni_2_N. The exposed surface with heterogenous interfaces of FeNi_3_ alloy and FeNi_3_N/Fe_2_Ni_2_N enabled effective electronic structure manipulation via phase control (Figure 8C). According to the calculated *d*‐band center, the (FeNi_3_N, Fe_2_Ni_2_N)/FeNi_3_ was neither too high nor too low, showing the lowest theoretical overpotential (Figure 8D). High *E*_d_ energy levels strengthen the bonds too much, making oxygen desorption difficult, while low *E*_d_ energy levels weaken the bonds too much. The dual‐phase nitrides accelerated the overall OER kinetics with surfaces of appropriate adsorption energy [123]. Meanwhile, as the constituent metal elements increase, the surface oxide layer formed in the OER environment undergoes more complex chemical changes [124]. Hence, it is necessary to thoroughly examine the relationship between structural change and the activity of multi‐component nitrides. Yuan et al. found that after OER of Co_3_Mo_3_N, an amorphous oxide and hydroxide layer of several nm formed on the surface, with Co becoming richer near the top of this surface oxide layer (Figure 8E) [125]. Mo atoms move to the inner bulk, forming short bonds with N atoms to compensate for the dangling bonds on the surface, while Co atoms on the surface are further oxidized to provide favorable valence for OER (Figure 8F). In addition, the substitution of the Fe in the Co site of Co_3_Mo_3_N changed the reconstructed layer to contain more OER‐active Co^3+^. Co_2.5_Fe_0.5_Mo_3_N exhibited a low Tafel slope of 41 mV dec^−1^, which is approximately half that of Co_3_Mo_3_N [126]. Each metal element adapts differently to the oxidizing environment and influences each other closely.

Ternary and quaternary nitrides have also been developed for seawater splitting, which is much harsher than conventional OER. During the seawater electrolysis, OER and chlorine evolution reaction (CER) compete at the anode [127]. According to pH change and applied potential, various side reactions, such as chlorine precipitation, hypochlorous acid formation, and hypochlorite formation, occur. In particular, since the hypochlorous acid and hypochlorite are preferentially formed during CER involving two electrons when the local pH is low, the material properties deteriorate, so a material that can effectively lower the overpotential of OER is required. Yu et al. developed a 3D core‐shell metal nitride consisting of NiFeN nanoparticles on NiMoN nanorods supported on Ni foam [128]. The NiFe nanoparticles in situ evolve NiFe (hydro)oxide during OER, requiring very low overpotentials of 369 and 398 mV to deliver current densities of 500 mA cm^−2^ and 1 A cm^−2^ in alkaline natural seawater. Similarly, Wang et al. introduced cobalt‐molybdenum nitride catalysts for seawater oxidation [129]. From the ZIF‐67‐derived Mo–Co precursors, MoN–Co_2_N nanosheets were synthesized, providing active catalytic performances for both OER and HER. The bifunctional catalyst recorded a low voltage of 1.7 V to achieve a current density of 100 mA cm^−2^ for overall seawater splitting in the Mediterranean Sea. Ma et al. utilized heterointerfaces of FeCoP and Ni_3_N for the overall seawater splitting [130]. The 3D heterogeneous structure of (Fe_1−_ *_x_* Co*_x_*)_2_P/Ni_3_N electrocatalyst provided abundant active sites and enhanced electrical conductivity, accelerating charge transfer between phosphide and nitride. For overall alkaline seawater electrolysis, the heterogeneous catalyst only required a voltage of 1.645 V to generate a current density of 100 mA cm^−2^.

Instead of using metal nitrides’ spontaneous oxidation surface, designing heterostructure has also become a prominent strategy [131, 132]. For example, NiMoN/NiFe layered double hydroxides (LDH), which was electrodeposited NiFe LDH on Ni_0.2_Mo_0.8_N nanorods, showed an overpotential of only 266 mV to reach a high current density of 1 A cm^−2^ and stability of 250 h. This remarkable property is attributed to the formation of more active Ni^3+/4+^ at lower potentials in NiMoN/NiFe LDH, as revealed by in situ Raman analysis. In addition, EIS analysis by applied potential verifies the acceleration of interfacial charge transfer and rapid HO^*^ adsorption kinetics in the heterostructure [133]. Recently, metal nitride has been introduced as a catalyst material for use in proton exchange membrane water electrolyzer (PEMWE). A study focusing on the functionality of the IrO*_x_*/TiN heterointerface revealed that Ir‐Ti bonding improves OER activity and stability. The Ir‐Ti interaction appropriately regulated the HO^*^ binding of IrO*_x_* and reduced Ir oxidation at the interface in an acidic electrolyte. Applied to PEMWE, the IrO*_x_*/TiN catalyst showed lower cell voltage than the commercial IrO_2_ catalyst [134]. Jeong et al. reported increased OER activity in IrO*_x_* synthesized on Fe_2_N support compared to IrO*_x_*/Pt (Figure 8G) [135]. The porous nanostructure of Fe_2_N reduced overall ohmic loss and mass transfer resistance in PEMWE. This suggests that metal nitrides, which function as a support and protective layer in PEMWE, can replace noble metals.

#### 4.1.3 Oxygen Reduction Reaction

Oxygen reduction reaction (ORR) serves as the cathode reaction in energy conversion devices such as fuel cells and metal‐air batteries. It proceeds through two different routes: a four‐electron pathway and a two‐electron pathway. In the 4*e* ^−^‐ORR, oxygen directly receives four electrons and is reduced to water. In the two‐step 2*e* ^−^‐ORR, oxygen is first reduced to H_2_O_2_ and then reduced to water [136]. The reaction path for each pH for each pH is shown below.

$$\text{In acidic media}:O_{2}+4H^{+}+4e^{-}\to 2H_{2}O\left(4e^{-}-\mathrm{ORR}\right)$$

$$O_{2}+2H^{+}+2e^{-}\to H_{2}O_{2}\;\left(2e^{-}-\mathrm{ORR}\right)$$

$$H_{2}O_{2}+2H^{+}+2e^{-}\to 2H_{2}O$$

$$\mathrm{In}\;\mathrm{alkaline}\;\mathrm{media}:O_{2}+2H_{2}O+4e^{-}\to 4{\mathrm{OH}}^{-}\;\left(4e^{-}-\mathrm{ORR}\right)$$

$$O_{2}+H_{2}O+2e^{-}\to {{\mathrm{HO}}_{2}}^{-}+{\mathrm{OH}}^{-}\left(2e^{-}-\mathrm{ORR}\right)$$

$${{\mathrm{HO}}_{2}}^{-}+H_{2}O+2e^{-}\to 3{\mathrm{OH}}^{-}$$

H_2_O_2_ formed during ORR is considered undesirable since it reduces efficiency and affects the stability of the proton exchange membrane. Recently, research has been performed to increase the selectivity of the reduction of O_2_ to H_2_O_2_ (2*e* ^−^‐ORR) due to the usefulness of H_2_O_2_ as a chemical fuel [137]. ORR catalyst materials still need to be developed as non‐Pt group materials that will surpass Pt, the performance benchmark. Metal nitrides are being developed as efficient and practical ORR catalysts through phase, composition, structure, and surface control.

**FIGURE 9:**
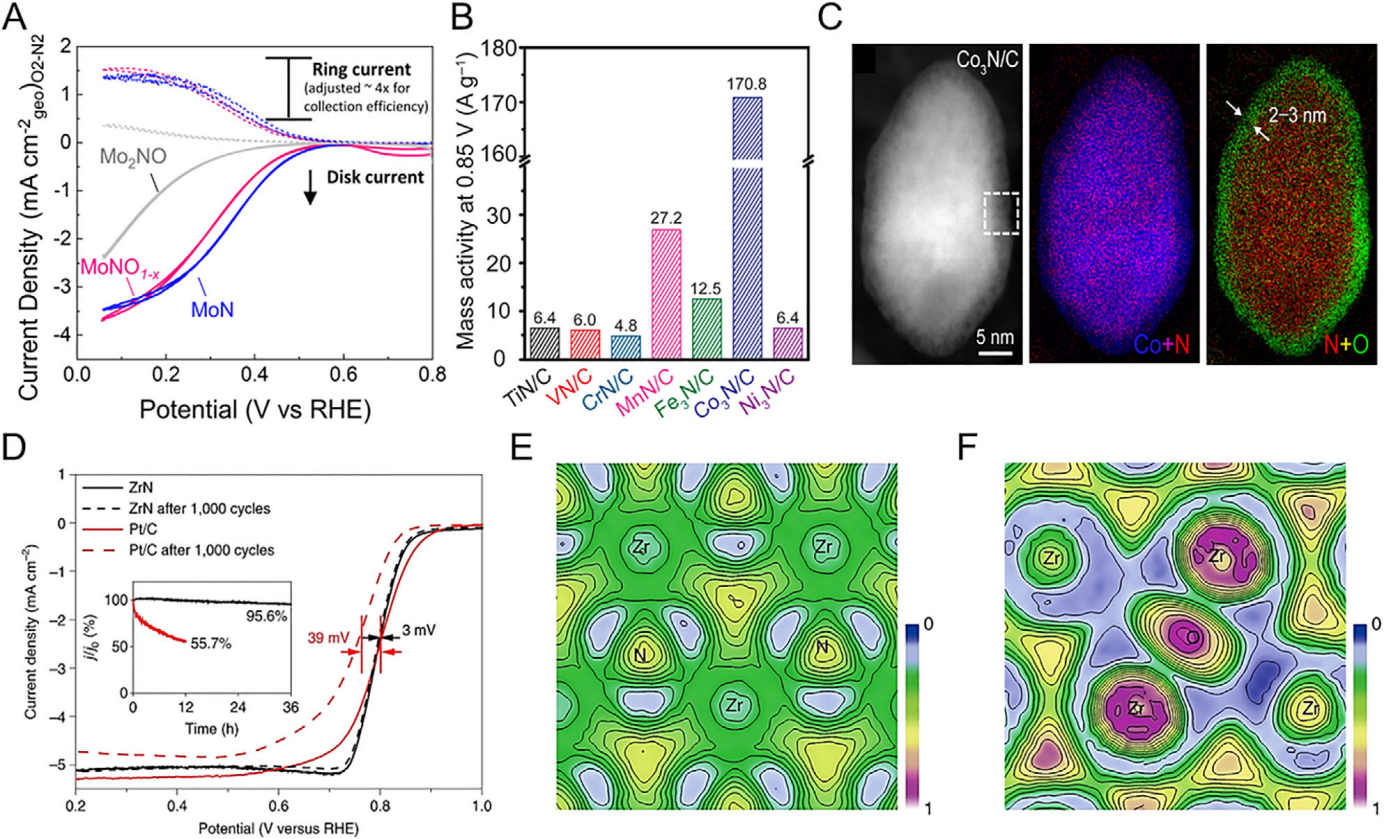
(A) Cyclic voltammograms of MoN, MoNO_1–_ *_x_*, and Mo_2_NO in O_2_‐saturated 0.1 M HClO_4_ electrolyte from the disk and the ring. Reprinted with permission [138]. Copyright 2020, American Chemical Society. (B) Comparison of the mass activity of all M*_x_* N/C measured at 0.85 V versus RHE in O_2_‐saturated 1 M KOH electrolyte. (C) HAADF‐STEM image of an elliptical Co_3_N particle and corresponding EELS elemental mapping of Co + N and N + O. Reprinted with permission [150]. Copyright 2022, AAAS. (D) *J*–*V* curves of ZrN and Pt/C before (solid curves) and after (dashed curves) the accelerated durability tests in O_2_‐saturated 0.1 M KOH solution. Inset: Chronoamperometric responses at a constant potential. Electron localization function (ELF) plots of the clean (E) and oxide‐adsorbed (F) surfaces of ZrN(111). Reprinted with permission [51]. Copyright 2020, Nature Publishing Group.

The ORR activity trend for the incorporation of oxygen, which can readily change the crystal structure of metal nitrides, has been reflected in the catalyst design. Kreider et al. investigated ORR activity and selectivity depending on oxygen inclusion in Mo‐based nitrides [138]. The contribution of 4*e* ^−^ and 2*e* ^−^‐ORR was confirmed by cyclic voltammetry using a rotating ring disk electrode (RRDE) for Mo_2_NO, MoNO_1 −_ *_x_*, and MoN with different bulk compositions (Figure 9A). MoN showed the highest ORR activity as the bulk oxygen content was minimized and a mixed hexagonal‐cubic structure developed. Additionally, bulk oxygen content had a dominant influence on 4*e* ^−^‐ORR, whereas it had a less sensitive effect on 2*e*−‐ORR. In addition, MnON with rock‐salt crystal structure also required appropriate O and N occupancy control [139]. As the nitrogen content increased in Mn−O, N octahedra, MnON had an almost single‐electron occupancy of *e*_g_ states, which was favorable for electron transfer of ORR [140]. To further exploit the electronic structure of oxynitride, ORR performance has been developed by adding structural modifications. For example, Co@W_0.62_(N_0.62_O_0.38_) with three‐dimensionally ordered macropores showed a half‐wave potential (*E*_1/2_) of 0.85 V versus reversible hydrogen electrode (RHE) in alkaline electrolyte, comparable to Pt of 0.86 V versus RHE [141].

For various metal compositions, nitrides have been explored for their ORR activity to develop materials with higher mass activity than Pt. In the early stages of developing materials to replace Pt groups, Cu_3_N nanocrystals showed promising ORR activity [142]. Cu_3_N has a ReO_3_‐type crystal structure with corner‐shared N‐centered Cu−N octahedra arranged in a cubic lattice. This structure allows for ternary compositions by inserting other metals into the central voids of the lattice. For example, Cu_3_PdN nanocrystals exhibited higher ORR activity than Cu_3_N and performance comparable to Pd. Additionally, Cu_3_PdN demonstrated superior mass activity and better alkaline operational stability than Pd [66]. Furthermore, ORR activity has also been investigated in other transition metal‐based nitrides such as Ni*_x_* N [143], Fe*_x_* N [144], and Co*_x_* N [145]. Insights from monometallic nitrides have led to the development of various bimetallic nitrides through precise control of different metal elements, including Nb_0.95_Co_0.05_N [146], V_0.95_Co_0.05_N [147], and Co_0.6_Mo_1.4_N_2_ [148]. Recently, the substitution of Co in (Co_0.17_Fe_0.83_)_3_N has been shown to manipulate the electronic configuration of Fe, thereby controlling the adsorption of intermediates. Ultimately, (Co_0.17_Fe_0.83_)_3_N anchored on N‐doped porous carbon reported excellent ORR activity with an *E*_1/2_ of 0.882 V versus RHE [149].

In a study by Zeng et al., the alkaline ORR characteristics of various non‐precious metal‐based nitrides (Ti, V, Cr, Mn, Fe, Co, Ni) were compared [150]. Among the candidates, Co_3_N/C, MnN/C, and Fe_3_N/C exhibited notable ORR activity. Exceptionally, Co_3_N/C showed the highest mass activity of 170 A g^−1^ and an *E*_1/2_ of 0.862 V, which is only 30 mV lower than that of Pt/C (Figure 9B). When exposed to the air, Co_3_N/C spontaneously forms a 2–3 nm oxide shell on its surface, providing an active surface and high conductivity (Figure 9C). This structure remains stable within the driving potential range below 1.0 V versus RHE. Further advancements in ORR activity were achieved by adjusting the Co/N ratio. Based on the observation that increasing the synthesis temperature leads to nitrogen loss and a higher Co/N ratio, Co_4_N/C was successfully synthesized. Co_4_N/C exhibited an *E*_1/2_ of 0.875 V versus RHE and a minimal decrease of only 14 mV after 10,000 cycles [151]. Nitride catalysts have made substantial advancements in various aspects, such as composition and morphology, nearly reaching the performance of Pt. Nevertheless, achieving superior performance compared to Pt remains challenging. However, Yuan et al. corroborated the excellent ORR performance of ZrN, opening the possibility of developing ORR catalyst materials that can replace Pt [51]. ZrN had the same *E*_1/2_ of 0.8 V versus RHE as Pt/C and showed higher durability than Pt/C, decreasing by only 3 mV after 1000 cycles under alkaline conditions (Figure 9D). As a result of surface electronic structure investigation to determine the structure‐activity relationship, the most stable Zr‐terminated ZrN (111) surface had an oxygen adsorption energy similar to that of the Pt (111) surface. Additionally, ELFs of the pristine and oxygen‐adsorbed surface of ZrN showed high electron localization in small Zr–O clusters on the oxygen‐absorbed surface while high delocalization at the nitride (Figure 9F). These studies highlight the potential of nitride catalysts and ongoing efforts to improve ORR performance and durability.

#### 4.1.4 Nitrogen Reduction Reaction

Nitrogen reduction reaction (NRR) converts nitrogen in the air into ammonia, a useful chemical and energy carrier. Electrochemical conversion is gaining attention as a sustainable alternative to the traditional Haber–Bosch process, which requires high heat, pressure, and large amounts of fossil fuels. The primary mechanisms of electrochemical NRR are the dissociative, associative, and Mars‐van Krevelen (MvK) pathways involving different intermediates. Like the Haber–Bosch process, the dissociative route involves breaking the N_2_ triple bond before hydrogenating the cleaved N radicals to form NH_3_. Thus, it requires substantial energy and is rarely observed. The associative route, common in electrocatalytic NRR, hydrogenates N_2_ without breaking its triple bond, with variations like distal, alternating, and enzymatic processes based on the hydrogenation sequence of N atoms. The MvK mechanism, used to interpret NRR in transitional metal nitride electrocatalysts, involves forming NH_3_ from the reduction of surface N‐atoms and subsequent N_2_ refilling of vacancies [152]. Metal nitrides modify the *d*‐band of transition metals by integrating nitrogen atoms, increasing the density of states near the Fermi level. This makes their electronic structure similar to that of Pt group metals, providing an effective alternative to noble metal‐based catalysts. Metal nitrides capable of the MvK reaction pathway and with favorable electronic structures are being developed to improve activity and selectivity for ammonia production [153].

**FIGURE 10:**
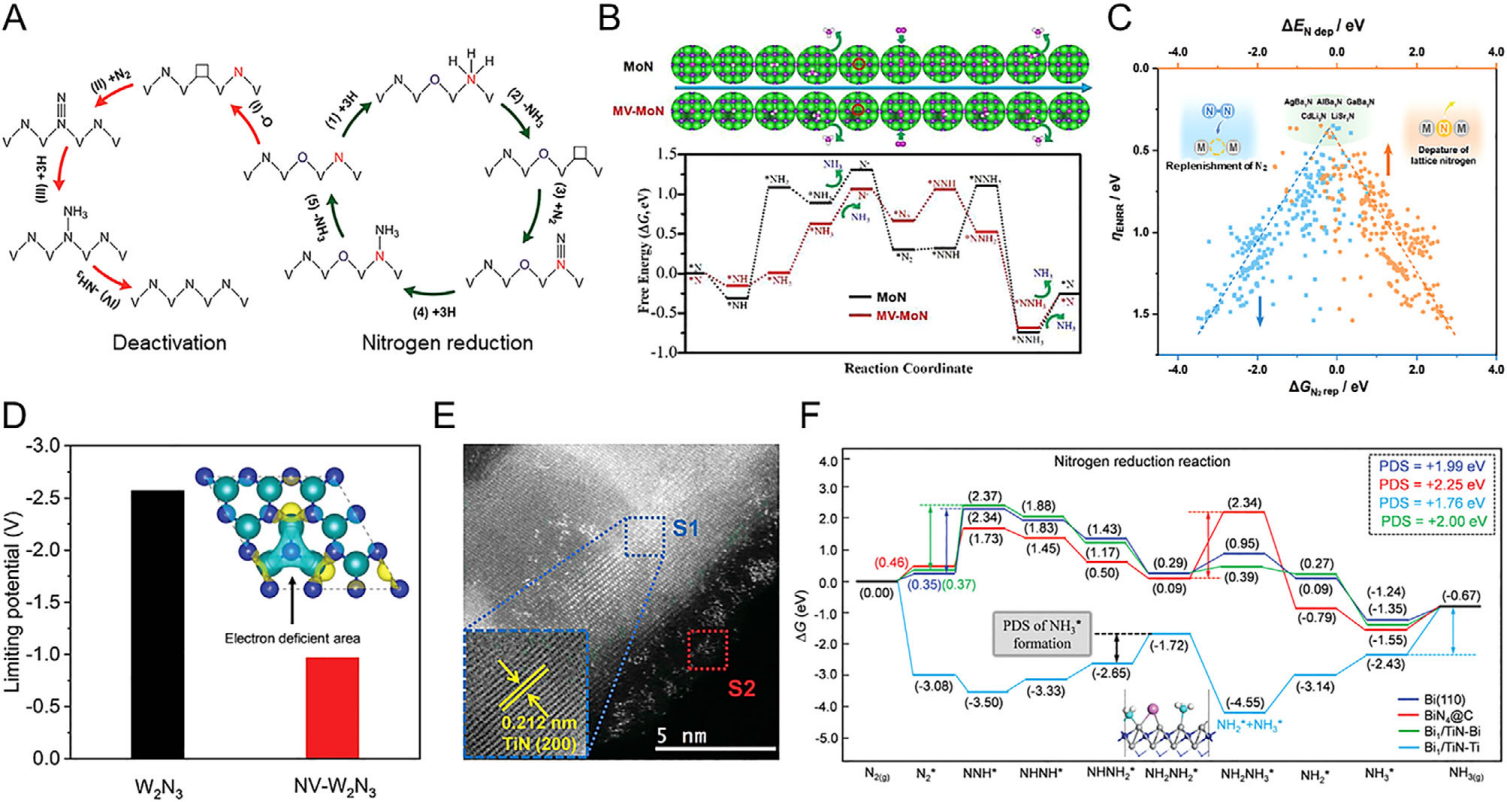
(A) Proposed reaction pathway for nitrogen reduction on the surface of VN_0.7_O_0.45_ via a Mars–van Krevelen mechanism and the catalyst deactivation mechanism. Reprinted with permission [156]. Copyright 2018, American Chemical Society. (B) Atomistic structure schemes and calculated Gibbs free energy evolution for NRR pathway on the (200) surfaces of defect‐free MoN and defect‐rich MV‐MoN, respectively. The green, purple, white, and red balls represent Mo, N from MoN, H, and N from N_2_, respectively. Reprinted with permission [159]. Copyright 2020, Elsevier. (C) Linear relation between Δ*G*_RDS_ and Δ*E*_N dep_ (upper *x*‐axis), Δ*G*_N₂ rep_ (lower *x*‐axis). Reprinted with permission [160]. Copyright 2023, Wiley‐VCH GmbH. (D) Magnitudes of the theoretical limiting potentials of NH_3_ production on W_2_N_3_ and NV‐W_2_N_3_. Inset: Charge density difference induced by nitrogen‐vacancy on VN‐W_2_N_3_. The value of iso‐surface is 0.005 e Bohr^−3^. Reprinted with permission [162]. Copyright 2019, Wiley‐VCH GmbH. (E) HAADF‐STEM image of NC/Bi SAs/TiN (the bright dots in the image represent single Bi atoms) (F) The free‐energy diagrams of NRR on the four different catalyst active sites of Bi (110), BiN_4_@C, Bi_1_/TiN‐Bi, and Bi_1_/TiN‐Ti at the electrode potential of 0 V. Reprinted with permission [163]. Copyright 2022, Wiley‐VCH GmbH.

Various metal nitride candidates for the MvK mechanism were initially screened using DFT calculations [154]. This process involved calculating the free energy of reaction pathways, identifying the rate‐determining and potential‐determining steps, and evaluating the kinetic and thermodynamic barriers for N vacancy diffusion into the bulk. Moreover, various factors, such as the possibility of catalyst poisoning, activity toward a competing HER, and the possibility of catalyst decomposition or regeneration, also required thorough verification. Abghoui et al. investigated the NRR catalysis via the MvK mechanism on group III–VII transition metal (Sc, Ti, V, Cr, Mn, Y, Zr, Nb, Mo, Hf, Ta, W, and Re) mononitrides using DFT calculations [155]. VN, CrN, MnN, NbN, and WN were computationally predicted to be active nitrides in NRR, with NbN proving to be a stable phase in the operational environment. Following the calculation results, NRR electrocatalysis of metal nitride was experimentally examined. In a study by Yang et al., VN nanoparticles exhibited NRR activity and selectivity, recording an NH_3_ yield of 3.3 × 10^−10^ mol s^−1^ cm^−2^ and a Faradaic efficiency (FE) of 6.0% at −0.1 V versus RHE [156]. To confirm the MvK mechanism of NRR electrolysis on the actual catalyst surface, an isotope labeling experiment in a ^15^N_2_ environment was performed. The simultaneous detection of ^14^NH_3_ and ^15^NH_3_ demonstrated that nitrogen in the catalyst surface participates in catalytic turnover. In addition, based on the findings that VN_0.7_O_0.45_ serves as an active phase, the reaction path via an MvK mechanism and deactivation path were proposed (Figure 10A). Surface nitrogen adjacent to oxygen serves as an active site for NRR, but the instability of the surface oxygen bond leads to deactivation and phase conversion to VN. A subsequent study of VN_0.7_O_0.45_ suggested that activation of adsorbed N_2_ on the N vacancy to adsorbed N_2_H is the rate‐limiting step and that surface N and N vacancies promote the catalyst turnover of NRR [157]. Identification of the active site through this elaborate isotope exchange experiment provided a guide to NRR catalyst design.

In Mo‐based nitride, Zhang et al. demonstrated the NRR activity of MoN nanosheets with an NH_3_ yield of 3.01 × 10^−10^ mol s^−1^ cm^−2^ and an FE of 1.15% at −0.3 V versus RHE [158]. Computational studies also revealed that MoN can catalyze NRR via the MvK mechanism. On the basis of MoN's catalytic activity, the influence of surface metal vacancy on NRR performance was examined. Mo‐vacancy‐rich MoN nanocrystals embedded in an N‐doped hierarchical porous carbon framework (MV‐MoN@NC) achieved a high NH_3_ yield of 76.9 µg h^−1^ ${\mathrm{mg}}_{\mathrm{cat}.}^{-1}$ at −0.2 V versus RHE, more than twice that of defect‐free MoN@NC. Additionally, MV‐MoN@NC demonstrated an FE of 6.9% and maintained long‐term stability for 48 h in 0.1 M HCl. DFT calculations revealed that Mo vacancies on the MoN surface significantly reduce the barrier height of the potential‐determining step (^*^NH_2_ + H^+^ + *e* ^−^ → ^*^NH_3_) from 1.40 to 0.61 eV, thereby enhancing NRR performance (Figure 10B) [159]. Furthermore, research has been done to understand the effects of changes in local chemistry within the lattice of various crystal structures. Zhang et al. studied the influence of changes in M' and M' elements of antiperovskite nitrides M'M_3_N on NRR catalysis via the lattice nitrogen‐participated MvK mechanism [160]. The descriptors proposed to explain the linear relationship with NRR activity in antiperovskite nitrides are the Gibbs free energy of the N_2_ replenishment process (Δ*G*_N₂ rep_) and lattice nitrogen departure energy (Δ*E*_N dep_) (Figure 10C). As a result, weak M─N bonds facilitate the involvement of lattice nitrogen and increase activity unless the M─N bonds are excessively weak, resulting in structural instability. The hybridization interactions between the M and N orbitals, as well as the M−M' polarization effect, both impact the M─N bond strength, which is crucial for activity. Through high‐throughput theoretical calculations, AgBa_3_N, AlBa_3_N, and GaBa_3_N have been identified as having selectivity for NRR.

Several studies have reported nitride catalysts that follow an associative mechanism rather than the MvK mechanism [161]. Jin et al. confirmed that 2D W_2_N_3_ nanosheets with nitrogen vacancies (NV‐W_2_N_3_) follow an associative distal mechanism through isotopic labeling experiments [162]. VN‐W_2_N_3_ showed a high NH_3_ yield of 11.66 ± 0.98 µg h^−1^ ${\mathrm{mg}}_{\mathrm{cat}.}^{-1}$ and an FE of 11.67 ± 0.93% at −0.2 V versus RHE for 24 h, stably maintaining a high ammonia production rate. Nitrogen vacancy as the active site formed an electron‐deficient environment, promoting N_2_ adsorption and lowering the limiting potential of the entire NRR process (Figure 10D). Meanwhile, single Bi atoms incorporated TiN nanorods encapsulated in a nitrogen‐doped carbon layer (NC/Bi SAs/TiN) demonstrated an associative alternating mechanism as a favorable NRR pathway (Figure 10E). This was observed for all four active sites: Bi(110), BiN_4_@C, Bi_1_/TiN‐Bi, and Bi_1_/TiN‐Ti (Figure 10F). According to the calculated reaction pathway, N_2_ molecules are rapidly hydrogenated from Ti sites of Bi_1_/TiN‐Ti to surface NH_3_. Subsequently, NH_3_ is desorbed from Bi sites of Bi_1_/TiN‐Bi and released between the carbon layer and Bi_1_/TiN. The effective route created by Bi and TiN's synergism highlights the versatility of catalytic surfaces in promoting the NRR process [163].

#### 4.1.5 Carbon Dioxide Reduction Reaction

Carbon dioxide reduction reaction (CRR) converts carbon dioxide into value‐added chemicals and fuels, addressing both carbon emissions and energy storage challenges. In terms of net‐zero carbon emission, electrochemical conversion is a sustainable alternative to traditional thermochemical processes, which are heavily reliant on fossil fuels. The primary mechanisms of electrochemical CRR include various reaction pathways that lead to different products, such as carbon monoxide (CO), formate (HCOO^–^), methane, ethylene (C_2_H_4_), and alcohol. The most common initial step in CRR is the reduction of CO_2_ to CO via a two‐electron transfer process. This pathway is particularly relevant for catalysts like gold and silver, which selectively produce CO. Further reduction of CO can lead to the production of multi‐carbon (C_2+_) products like ethylene and ethanol. Copper‐based catalysts have a moderate binding affinity for *CO intermediates, which allows further reduction into the C_2+_ products. Their reaction pathway proceeds through the formation of surface‐bound *CO intermediates, which then undergo C–C coupling to produce C_2+_ products.

**FIGURE 11:**
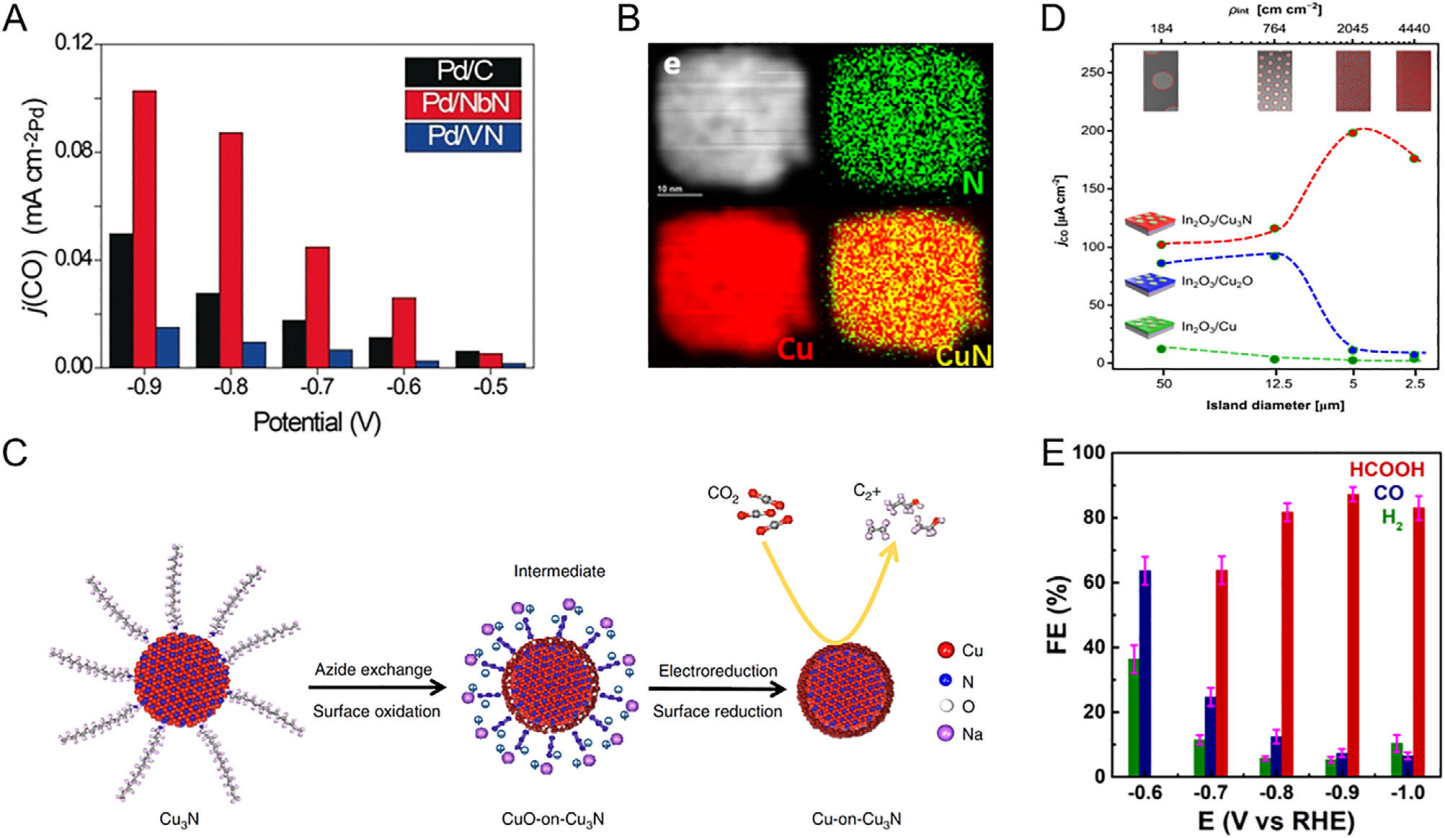
(A) Current densities of Pd‐modified transition metal nitrides for electrochemical CO_2_ reduction. Reproduced with permission [164]. Copyright 2020, Wiley‐VCH GmbH. (B) HAADF‐STEM image of Cu_3_N nanocube and the corresponding STEM‐EELS elemental mapping of the nanocube. Reproduced with permission [166]. Copyright 2019, American Chemical Society. (C) Schematic of preparing Cu‐on‐Cu_3_N catalyst. Reproduced with permission [167]. Copyright 2018, Springer Nature. (D) Electrocatalytic activity toward CO of microstructured multi‐component electrodes with different *ρ*_int_ controlled by preparing regular arrays of islands with different diameters and pitches. SEM images in which interfacial sites are highlighted in red are added for clarity. Reproduced with permission [169]. Copyright 2019, Wiley‐VCH GmbH. (E) Faradaic efficiencies of electrocatalysis using the InN‐C catalyst of 4.9 % In loading. Reproduced with permission [170]. Copyright 2021, Elsevier.

In addition to copper, metal nitride‐based catalysts have been explored for CRR due to their ability to modify the electronic structure of transition metals by integrating nitrogen atoms. The increased density of states near the Fermi level improves the catalytic activity to facilitate electron transfer during CRR. Also, the surface electronic properties of metal nitrides enable favorable binding energies for CO_2_ and reaction intermediates, leading to improved reaction kinetics and higher activity. Furthermore, as a support material, the high electrical conductivity of metal nitrides helps improve the overall electron flow during CRR, boosting the activity of the catalyst. Liu et al. studied the electrochemical CRR by supporting Pd catalysts on diverse transition metal nitride substrates [164]. As shown in Figure 11A, the Pd/NbN catalyst generated much higher CO partial current density and Faradaic efficiency than not only the Pd/VN but also the Pd/C catalyst. The active PdH phase on the NbN support stabilized *HCOO and weakened *CO intermediates, increasing CRR activity. Similarly, Pan et al. utilized the TiN support for the electrodeposition of Cu–Ni bifunctional electrocatalysts [165]. The high electrical conductivity of the TiN layer contributed to enhancing both CO_2_ reduction and oxygen evolution catalytic activity of Cu–Ni particles. The combined CRR–OER system recorded an overvoltage of 670 mV at 2 mA cm^−2^, exhibiting long‐term durability of 24 h.

As with metal catalysts, copper‐based nitrides are the most widely studied CRR catalyst among metal nitrides. The aforementioned moderate binding energy for intermediates makes Cu‐based nitrides suitable for producing C_2_ and C_3_ products. Yin et al. synthesized copper nanocubes using the precipitation method [166]. The Cu_3_N nanocube catalysts were formed from the *n*‐butylamine treatment of copper nanocubes, and the copper and nitrogen elements are homogeneously distributed within the nanocubes, as shown in Figure 11B. The Cu_3_N nanocubes showed high CRR selectivity and stability to C_2_H_4_ with a Faradaic efficiency of 60%. The stabilized Cu (I) sites by nitrogen in Cu_3_N are energetically favorable for CO–CHO coupling, enhancing the selectivity to C_2_H_4_. The heterointerface of copper metal and nitride has also been applied to C_2_ production. Liang et al. developed Cu‐deposited Cu_3_N CRR catalysts for C_2+_ production [167]. As depicted in Figure 11C, the Cu_3_N matrix was synthesized by the ligand exchange from octadecylamine to azide (N_3_^–^), and the electroreduction process by cyclic voltammetry formed an active Cu‐on‐Cu_3_N catalyst. Compared to Cu‐on‐Cu_2_O and Cu, Cu‐on‐Cu_3_N showed the lowest CO dimerization barrier energy and highest C_1_ pathway energy, exhibiting a Faradaic efficiency of 64% for C_2+_ products. Also, Cu_3_N supports effectively suppressed the reduction of Cu^+^, causing higher selectivity for C_2+_ formation. Similarly, Ebaid et al. formed a Cu catalyst by electrochemical reduction of thermally nitride Cu foil. The Cu_3_N‐derived Cu increased the surface roughness and undercoordinated sites, and the heterogeneous catalyst exhibited a high Faradaic efficiency of 68% in C_2+_ products. The improvement in selectivity of CRR catalysts according to nitride support also appeared in the C_1_ product. Veenstra et al. compared In_2_O_3_ catalysts with different copper‐based supports (Cu, Cu_3_N, and Cu_2_O) [169]. Figure 11D presents partial CO current densities with different supports according to In_2_O_3_ island diameter. In the case of oxide‐derived copper, the fast diffusion of indium rapidly formed interfaces. However, a metastable nitrogen species resulted in unchanging interfaces in nitride‐derived copper, causing the high selectivity of the CRR catalyst. The In_2_O_3_/Cu_3_N exhibited a Faradaic efficiency of 80% for CO and long‐term stability for 50 h. Indium also has high CRR activity even in its nitride phase. Hou et al. synthesized InN on carbon by the impregnation method and direct nitridation at 600°C [170]. The nitridation process forms more active sites, leading to increased catalytic activity and selectivity for CO of InN‐C compared to the In_2_O_3_‐C catalyst. As shown in Figure 11E, the maximum Faradaic efficiency reached 87% at –0.9 *V*_RHE_ for CO, and in MEA electrolysis, it recorded the highest value of 92.5%.

#### 4.1.6 Biomass Oxidation Reaction

Biomass oxidation reaction (BOR) converts biomass byproducts such as glycerol, glucose, methanol, ethanol, and 5‐hydroxymethylfurfural (HMF) into high‐value‐added chemicals. In addition to the economic advantage of high net value, BOR offers thermodynamic advantages due to lower oxidation potentials of organic molecules compared to OER. Recently, more BOR electrocatalysts have been developed in combination with hydrogen production, emphasizing low power consumption and high target product selectivity [171]. Since BOR and OER are both nucleophilic reactions, OER‐effective electrocatalysts exhibited catalytic activity in BOR as well. However, organic molecules consist of a wider variety of functional groups, which necessitates effective processes for adsorption and complex mass transfer to enhance selectivity for target products. Therefore, appropriate catalyst designs suitable for each biomass reactant are necessary.

**FIGURE 12:**
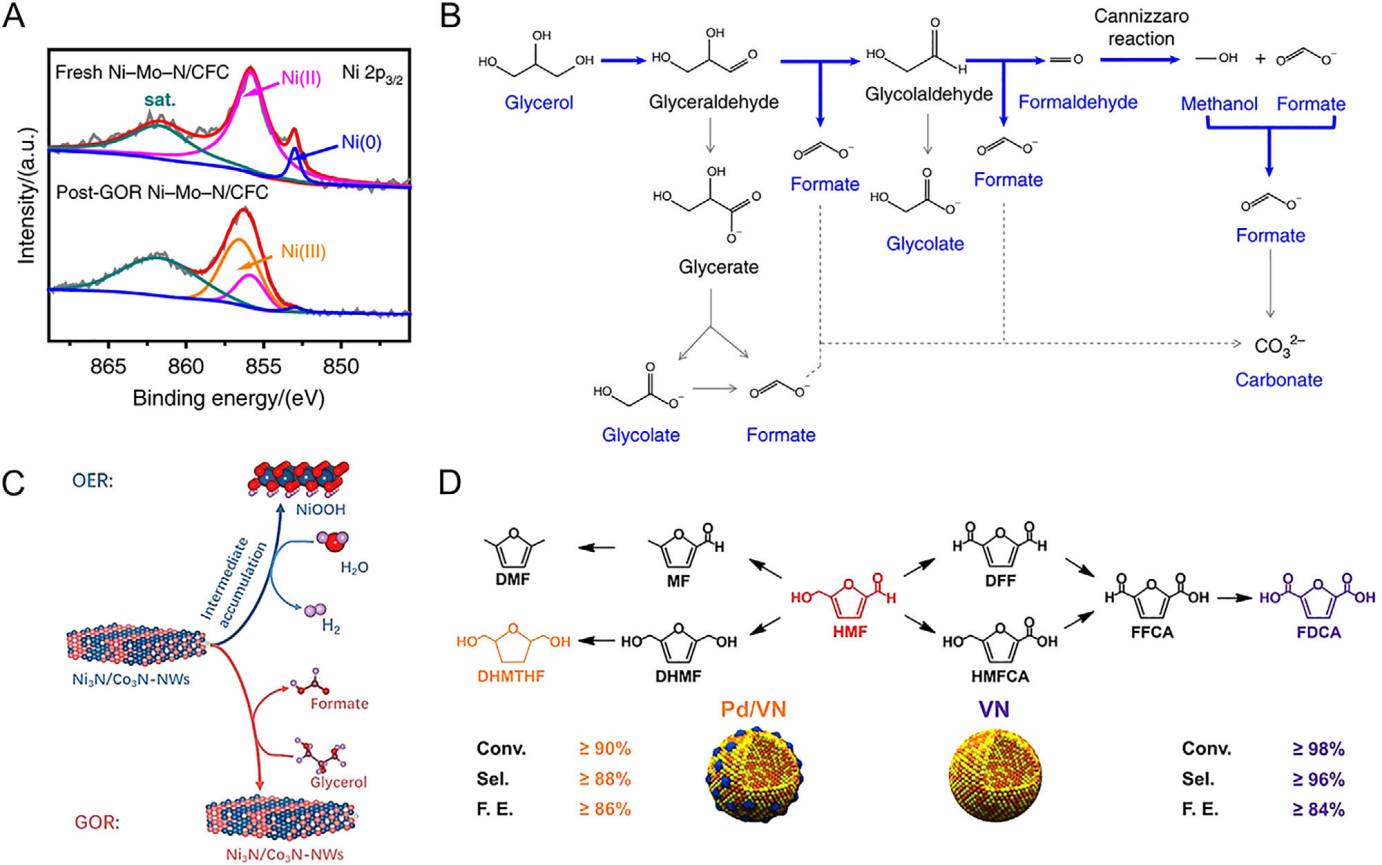
(A) Ni 2p_3/2_ XPS spectra of Ni–Mo–N/CFC before and after glycerol oxidation. (B) Proposed mechanistic scheme of glycerol electro‐catalytic oxidation to formate on Ni–Mo–N in alkaline medium (blue arrows is proposed to be the dominant pathways. Reprinted with permission [172]. Copyright 2019, Nature Publishing Group. (C) Schematic illustrations of the spontaneous glycerol electrocatalytic oxidation on the surface of Ni_3_N/Co_3_N‐NWs. Reprinted with permission [31]. Copyright 2023, Wiley‐VCH GmbH. (D) Electrocatalytic oxidation and electrocatalytic hydrogenation of HMF over VN and Pd/VN electrocatalysts, respectively. Reprinted with permission [176]. Copyright 2019, Wiley‐VCH GmbH.

Glycerol has a symmetrical chemical structure with three hydroxyl functional groups (−OH). Glycerol oxidation reaction (GOR) selectively electrooxidizes the primary and secondary hydroxyl groups in glycerol, producing C_3_ and C_2_ organic compounds containing carboxylic acids, phenolic groups, ketones, and aldehydes. Electrocatalytic GOR produces different final products depending on the operating pH and applied potential. Non‐precious metal‐based electrocatalysts have predominantly reported formate as the major product. In a study by Li et al., nickel‐molybdenum‐nitride nanoplates loaded on carbon fiber cloth (Ni‐Mo‐N/CFC) containing Ni_0.2_Mo_0.8_N phase showed a decreased anodic potential of 1.30 V versus RHE at a current density of 10 mA cm^−2^ in 1 M KOH with 0.1 M glycerol, which was 270 mV lower than that without glycerol [172]. Investigation into the high activity of Ni‐Mo‐N/CFC through XPS analysis after GOR revealed that Ni (0) and Ni (II) were oxidized to NiOOH, reported as active species in glycerol oxidation (Figure 12A). Post‐GOR analysis also confirmed that surface Mo dissolution creates surface defects that enhance activity, while Mo species do not serve as active sites for GOR. Moreover, the GOR mechanism in Ni‐Mo‐N/CFC with high formate conversion efficiency was elucidated using isotopic labeled nuclear magnetic resonance (NMR) spectroscopy. After the oxidation of glycerol to glyceraldehyde, C–C bond cleavage of glyceraldehyde, rather than sequential oxidation through glycerate, was found to be the dominant route for formate formation (Figure 12B). Recently, Co‐based nitrides also showed improved GOR activity, such as carbon shell‐encapsulated Mn‐doped cobalt nitride nanoarrays (Mn‐CoN@C) [173] and CuCoN_0.6_/CP [174]. CoOOH formed by surface oxidation generally acts as an active site, and a dominant pathway for formate formation via C─C bond cleavage in glycerate has been proposed. Meanwhile, Ni_3_N/Co_3_N‐NWs demonstrate integrated OH^*^‐involved direct oxidation and oxyhydroxide‐involved indirect oxidation in GOR mechanisms through in situ Raman spectroscopy and operando synchrotron‐radiation Fourier transform infrared spectroscopy (SR‐FTIR) confirmation (Figure 12C). Additionally, DFT calculations demonstrated that the Ni_3_N/Co_3_N‐NW‐based heterointerface modulates the electron distribution and accelerates the glycerol dehydrogenation kinetics [31].

HMF oxidation reaction (HMFOR) has two possible pathways to produce 2,5‐furandicarboxylic acid (FDCA) from HMF, which has a simple molecular structure containing a furan ring, –C_2_O, and –OH groups. The first is the oxidation of the aldehyde to the carboxyl group to form the intermediate 5‐hydroxymethyl‐2‐furancarboxylic acid (HMFCA). The second is the oxidation of the hydroxyl group to an aldehyde group to form 2,5‐diformylfuran (DFF). Both HMFCA and DFF are then oxidized to 2‐formyl‐5‐furancarboxylic acid (FFCA) and finally to FDCA. Depending on the electrolyte's pH, the first route is dominant in strongly alkaline at pH above 13, and the second route is dominant at pH below 13 [175]. HMFOR catalysts exhibit high activity even in non‐precious metal‐based materials, with most showing nearly 100% FDCA conversion efficiency. However, achieving high current density at low potentials, as well as increasing current density values, remain challenges for catalyst materials. The HMF oxidation activity of nitride catalysts was first reported in VN. The VN electrocatalyst required 1.36 V versus RHE to reach 10 mA cm^−2^ in 1 M KOH with 10 mM HMF, demonstrating an HMF conversion rate of 98%, a selectivity of 96%, and an FDCA FE of 84%. Theoretical calculations revealed that the higher performance of VN, compared to V_2_O_5_, is due to its closer *d*‐band center to the Fermi level, which facilitates HMF adsorption and activation. Furthermore, a paired biomass upgrading system was designed with VN as the anode and Pd‐decorated VN (Pd/VN) as the cathode (Figure 12D). This demonstrated the simultaneous production of FDCA and 2,5‐bishydroxymethyl‐tetrahydrofuran (DHMTHF) through HMF oxidation and hydrogenation processes, respectively [176]. High HMFOR activity has also been identified in Ni‐based nitrides, coupled with characterization of the catalytic active sites. For example, carbon‐coupled nickel nitride nanosheets (Ni_3_N@C) reported a low potential of 1.38 V versus RHE to achieve a current density of 50 mA cm^−2^ in 1 M KOH with 10 mM HMF, with an FDCA FE of 98% [177]. A subsequent study using operando X‐ray absorption spectroscopy (XAS) and in situ Raman spectroscopy has provided insights into the electrocatalytic behavior of Ni_3_N. The HMFOR process of Ni_3_N is described as a proposed two‐step reaction. Initially, Ni atoms lose electrons and adsorb OH ions under applied potential, forming NiN(OH)_ads_. Then, the electrophilic oxygen in NiN(O)_ads_ interacts with HMF to generate water and promote the reaction. According to this activation mechanism, increasing electrolyte pH enhances HMFOR activity by increasing the number of active species [178].

### 4.2 Photoelectrocatalysis

Photoelectrocatalysis is distinguished from electrocatalysis by using photo‐excited electrons and holes of semiconductor photoelectrodes to drive electrochemical conversion reactions. The photoelectrochemical (PEC) process involves a series of steps, including light absorption, separation and transport of photogenerated charge carriers, and injection of these charge carriers [179]. Fundamentally, PEC performance depends on the intrinsic properties of the light absorber, such as bandgap, band position, and carrier mobility. The band gap is one of the most critical factors in determining photocurrent density and photovoltage, with the ideal value ranging from 1.8 to 2.2 eV [180]. Metal (oxy)nitrides, which can narrow the bandgap through high N 2*p* orbitals compared to oxides, are considered promising materials active in the visible light spectrum. In addition, charge transport layers, co‐catalysts, and surface passivation layers have been developed to minimize recombination and increase PEC efficiency. Metal nitrides also play a crucial role as surface overlayers, providing various functionalities from their intrinsic electronic structure. Studies in which metal nitrides were used as light absorbers and functional layers in photoelectrocatalysis are summarized in Table 2.

**TABLE 2:** Photoelectrochemical performance of metal nitride catalysts for various catalytic reactions.

Oxynitride				
Catalysts	Electrolytes	*E*_onset_ (V vs. RHE)	*J*_ph_ *@* 1.23 V vs. RHE (mA cm^−2^)	Stability (min)
CoO*_x_*/LaTiO_2_N/Ta/Ti [182]	0.1 M NaOH	–	8.9	120
CoOOH/SrTaO_2_N single crystal [183]	1 M NaOH	0.35	1.8	–
NiCoFe‐B_i_/BaTaO_2_N/Nb [184]	1 M KOH	0.55	4.7	10
Co(OH)*_x_*‐FeO*_x_*/BaNbO_2_N/Ta/Ti [186]	0.5 M KB_i_ (pH 13)	–	5.2	2
IrO_2_/LaTiO*_x_* N*_y_*/TiN/MgO [188]	0.5 M NaOH	–	0.003	–
Co(OH)*_x_*‐FeO*_x_*/BaTaO_2_N/Ta/Ti [189]	0.5 M KB_i_ (pH 13)	0.6	6.5	1440

Tantalum nitride				
Catalysts	Electrolytes	*E*_onset_ (V vs. RHE)	*J*_ph_ *@* 1.23 V vs. RHE (mA cm^−2^)	Stability (min)
TiO_2_/Ta_3_N_5_/Ta [191]	1 M NaOH	0.95	2.46	–
Ni_0.50_Fe_0.50_O*_x_*/Ta_3_N_5_/Ta [193]	1 M NaOH	0.6	9.80	180
N:CoFeO_x_/Ta_3_N_5_/NbN*_x_*/Si [194]	1 M KOH	0.27	9.28	10
FeNiO*_x_*/Ta_3_N_5_ nanorods/Ta [77]	0.5 M KP_i_ (pH 13)	0.57	9.95	70
Ta_3_N_5_/FTO [195]	0.1 M K_4_[Fe(CN)_6_] (pH 7)	0.3	2.4	–
FeNiO_x_/BaTaO_2_N/Ta_3_N_5_ nanorods/SiO_2_ [197]	1 M KP_i_ (pH 13)	0.6	4.7	75
Co‐P_i_/Ba:Ta_3_N_5_ nanorods/Ta [199]	0.5 M KP_i_ (pH 13)	0.6	6.7	20
NiCoFeO*_x_*/Ta_3_N_5_ nanorods/GaN/Al_2_O_3_ [200]	1 M KOH	0.75	10.8	45
NiCoFe‐B_i_/Mg:Ta_3_N_5_/NbN_x_ nanorods [201]	1 M KOH	0.46	7	150
CoOOH/Mg:Ta_3_N_5_/FTO [202]	1 M NaOH	0.6	6.5	70
Co(OH)*_x_*/Zr:Ta_3_N_5_/Ta [203]	1 M KOH	0.51	7.2	5
NiCoFe‐B_i_/La:Ta_3_N_5_/Mg:Ta_3_N_5_/Nb [204]	1 M KOH	0.39	10.06	120
Co(OH)*_x_*/Sc:Ta_3_N_5_/FTO [205]	1 M NaOH	0.4	4.9	120
NiCoFe‐B_i_/gradient‐Mg:Ta_3_N_5_/Nb [207]	1 M KOH	0.57	8.5	300
NiFeO_x_/CPF‐TTB/Ta_3_N_5_/TiN/Al_2_O_3_ [30]	1 M KOH	–	9.12	10
NiFeO*_x_*‐CH_3_CN/Ta_3_N_5_ nanorods/Ta [208]	1 M KOH	0.7	6.2	120
NiFeO*_x_*/Ta_3_N_5_/GaN/Al_2_O_3_ [209]	1 M KOH	0.6	7.4	80

III‐nitride						
	**Catalysts**		**Electrolytes**	***E*_onset_ (V vs. RHE)**	***J*_ph_ (mA cm^−2^)**	**Stability (h)**
Light absorber	OER	IrO*_x_*/InGaN nanowires [211]	0.5 M H_2_SO_4_	0.1	10.9 @ 1.23 V vs. RHE	0.5
	HER	Pt/Al_2_O_3_/*p^+^*‐InGaN/ *n* ^++^/*p* ^++^InGaN/*n* ^+^‐*p* Si [212]	0.5 M H_2_SO_4_	–	9 @ 0.3 V vs. RHE	100
	**Catalysts**		**Electrolytes**	**Performance**		**Stability (h)**
		Rh/Cr_2_O_3_/GaN nanowires [213]	Neutral water	Quantum efficiency = 0.5%		–
	Overall water splitting	Rh‐CoO*_x_*/GaN nanorods [214]	1 M NaOH	H_2_ evolution = 1.4 mol h^−1^ g^−1^, Quantum efficiency = 6.9%		3
		Rh/Cr_2_O_3_/*p*‐GaN/*p*‐In_0.2_Ga_0.8_N [215]	Neutral water	H_2_ evolution = 3.46 mol h^−1^ g^−1^, Quantum efficiency = 12.3%		–
		Rh/Cr_2_O_3_/Co_3_O_4_/InGaN/GaN [216]	Neutral water (70°C)	STH efficiency = 9.2%		2.3

	Catalysts	Electrolytes	*E*_onset_ (V vs. RHE)	*J*_ph_ (mA cm^−2^)	Stability (h)
Functional layer	Nonpolar sidewalls exposed‐GaN/*n* ^+^‐*p*‐Si [217]	0.5 M H_2_SO_4_	0.45	18 @ 0 V vs. RHE	2
	*n*‐GaN nanowires/*n* ^+^‐*p*‐Si [218]	0.5 M H_2_SO_4_	0.36	30 @ 0 V vs. RHE	3000
	Pt nanoclusters/GaN/*n* ^+^‐*p*‐Si [219]	0.5 M NaCl	0.4	33 @ ‐0.4 V vs. RHE	120
	MoS*_x_* @GaN/n^+^‐*p*‐*p* ^+^‐Si [220]	0.5 M H_2_SO_4_	0.4	40 @ 0 V vs. RHE	10

*Note*: E_onset_ refers to the onset potential; *J*_ph_ refers to the photocurrent density.

#### 4.2.1 Oxynitride

**FIGURE 13:**
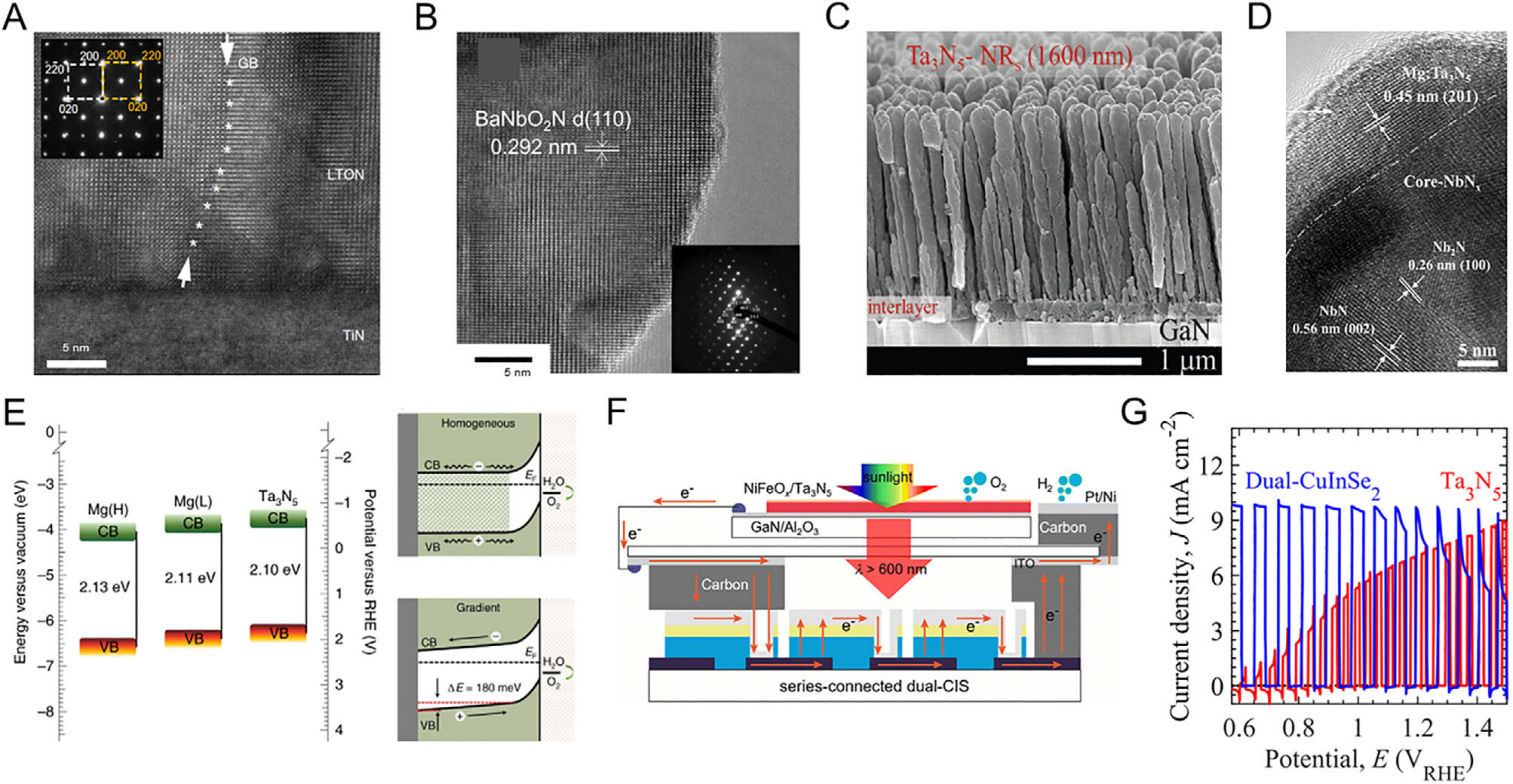
(A) HAADF‐STEM image of LaTiO_2_N‐TiN film grown on MgO. Inset: corresponding SAED pattern of LaTiO_2_N. Reprinted with permission [188]. Copyright 2020, Nature Publishing Group. (B) HR‐TEM image of BaNbO_2_N (L) particles as‐prepared by nitridation at 1173 K for 20 h and after subsequent annealing Ar at 873 K for 1 h. Inset: Corresponding SAED pattern of BaNbO_2_N. Reprinted with permission [186]. Copyright 2018, Wiley‐VCH GmbH. (C) SEM image of Ta_3_N_5_‐NRs/Ta_3_N_5_‐thin film/GaN. Reprinted with permission [200]. Copyright 2023, Wiley‐VCH GmbH. (D) HR‐TEM image of the NbN*_x_* @Mg:Ta_3_N_5_‐NR samples. Reprinted with permission [201]. Copyright 2023, Wiley‐VCH GmbH. (E) Energy band diagrams of pristine Ta_3_N_5_, Mg(L):Ta_3_N_5_, and Mg(H):Ta_3_N_5_ films and band bending schematics of homogeneous and gradient Mg:Ta_3_N_5_ photoanodes. Reprinted with permission [207]. Copyright 2020, Nature Publishing Group. (F) Schematic diagram of a tandem cell, composed of NiFeO*_x_*/Ta_3_N_5_/GaN/Al_2_O_3_ photoanode, dual‐CuInSe_2_ PV cells, and Pt/Ni electrode. (G) *J*–*E* curves of Ta_3_N_5_ photoanode (in front) and dual‐CuInSe_2_ PV cells (behind photoanode). Reprinted with permission [209]. Copyright 2022, Royal Society of Chemistry.

Prior to the development of nitride photoelectrodes, photoelectrodes using perovskite‐type oxynitrides as light absorbers were introduced. Perovskite‐type oxynitrides have an AB(O, N)_3_ structure, where A is a large alkali‐earth metal (Ca, Sr, or Ba) or La and B is a relatively small transition metal (Ti, Ta, or Nb). In the synthesis process of oxynitrides from oxides, the valence band maximum is determined by the hybridization of N 2*p* and O 2*p* orbitals, narrowing the band gap of the oxides. The conduction band minimum consists of Ti 3*d*, Nb 4*d*, or Ta 5*d* orbitals with a *d* ^0^ electronic configuration, and its position is determined by the tilt of B(O, N)_6_ octahedra [181]. The band gap can be adjusted between 1.8 and 2.5 eV depending on the A and B cations combination. Representatively, visible light‐active n‐type oxynitrides have been developed, including LaTiO_2_N (*E*_g_ = 2.1 eV) [182], SrTaO_2_N (*E*_g_ = 1.8 eV) [183], BaTaO_2_N (*E*_g_ = 1.8 eV) [184], SrNbO_2_N (*E*_g_ = 1.9 eV) [185], and BaNbO_2_N (*E*_g_ = 1.7 eV) [186]. To investigate the practical use of these photoanode materials, various research groups have focused on their photoactivity and stability. The Lippert group studied the photoactive facet [187] and the surface structural stability [188] during PEC water oxidation of LaTiO_2_N, epitaxially grown by pulsed laser deposition on TiN/MgO (Figure 13A). They observed surface modifications at the interface with the electrolyte, confirming the necessity of a co‐catalyst layer to ensure operational stability. In addition, most studies have fabricated photoelectrodes from oxynitride particles that achieve a crystalline phase after the nitridation of an oxide‐based precursor. Simultaneously, surface control techniques such as post‐thermal treatment and surface etching have been developed to reduce charge recombination, significantly enhancing PEC performance [182, 189]. For example, Seo et al. improved the crystallinity of BaNbO_2_N through post‐inert Ar annealing, suppressing the formation of reduced Nb species that cause surface defects and impurity phases (Figure 13B) [186]. The synthesized BaNbO_2_N was fabricated into photoelectrodes using the particle transfer method, achieving a photocurrent density of 5.2 mA cm^−2^ at 1.23 V versus RHE with the aid of a Co(OH)*_x_*‐FeO*_x_* co‐catalyst.

#### 4.2.2 Tantalum Nitride

The higher potential energy of the N 2*p* than O 2*p* orbital raised expectations that the development of narrow band gap transition metal nitrides could surpass the performance limitations of oxides. However, most transition metal nitrides exhibit metallic properties, and Ta_3_N_5_ has been the most extensively studied of the few materials possessing semiconductor properties. Ta_3_N_5_, an n‐type semiconductor, has a band gap of 2.1 eV and a band position that straddles the water‐splitting level, which meets the requirements for a photoanode [190]. Ta_3_N_5_ is primarily synthesized by nitriding an oxide‐based precursor in a gaseous ammonia atmosphere, and various methods for synthesizing oxide precursors have been reported. The synthesis of Ta‐based oxide involves methods such as thermal oxidation [191], anodization [192], and hydrothermal [193] synthesis using Ta foil, as well as deposition techniques like e‐beam evaporation [194], sputtering [77], and ALD [195]. Additionally, recent reports have shown the synthesis of Ta_3_N_5_ using Ta [196] and TaON [197] as precursors. The subsequent nitridation of the precursor typically occurs at temperatures ranging from approximately 800 to 1000°C. These nitridation conditions make it difficult to use oxide‐based transparent electrodes and require careful selection of the substrate. Moreover, this synthesis route inevitably includes point defects such as reduced Ta^3+^ and nitrogen vacancies, which act as deep‐level trap sites causing charge carrier recombination and thereby reducing PEC performance [198].

Nanostructuring is a representative engineering strategy applied to Ta_3_N_5_ as it increases light absorption and shortens the charge transfer path. Early work on the synthesis of 1D nanostructures focuses primarily on anodization. Ba:Ta_3_N_5_ NRs produced by mask anodization of Ta foil exhibited 6.7 mA cm^−2^ at 1.23 V in an alkaline electrolyte with a Co‐Pi co‐catalyst [199]. Subsequently, nanorod structures using sputtering GLAD have also made significant progress. Pihosh et al. successfully synthesized a transparent nanostructured photoanode on a GaN/Al_2_O_3_ substrate using GLAD, consisting of a 150 nm Ta_3_N_5_ thin film interlayer and 1.6 µm Ta_3_N_5_ NRs (Figure 13C) [200]. Optoelectrical simulation of this structure showed that it can achieve a photocurrent density of 10.5 mA cm^−2^ with a charge diffusion length of 99 nm, attributed to the high light absorption of the nanostructure and minimal reflection. This result is similar to the current density of 10.8 mA cm^−2^ at 1.23 V versus RHE obtained experimentally in the final structure incorporating FeNiCoO*_x_* co‐catalyst. These findings highlight the importance of optimizing nanostructure design to reduce recombination and increase current density. Another recently reported notable nanostructured photoelectrode is the core‐shell NbN*_x_* @Mg:Ta_3_N_5_ NRs (Figure 13D) [201]. This structure was obtained by depositing Mg‐doped TaO*_x_* on Nb_2_O_5_ NRs by plasma enhanced‐ALD and nitriding it. Photogenerated electrons from the Mg:Ta_3_N_5_ shell transfer efficiently to the highly conductive NbN_x_ nanorods, improving charge separation. Even with a Ta_3_N_5_ shell thickness of less than 30 nm, the photoanode exhibited a high photocurrent density of 7 mA cm^−2^ at 1.23 V versus RHE, modified with NiCoFe‐B_i_ co‐catalyst.

Doping is also a main strategy for improving the intrinsic properties of Ta_3_N_5_ semiconductors. Dopants such as Ba [199], Mg [202], Zr [203], La [204], and Sc [205] have been used in Ta_3_N_5_ for various functions, including enhancing electronic mobility, narrowing the band gap, and suppressing defects. For example, Zr^4+^, which enters Ta^5+^ as a compensating electron acceptor, increases charge conductivity by eliminating deep‐level defects related to Ta^3+^. Ti^4+^, with an ion radius more similar to Ta^5+^, performs a role similar to Zr^4+^ while minimizing changes in lattice parameters that would widen the band gap [206]. The dopant's valence state, ion radius, oxygen affinity, and other factors collectively influence the structure and PEC performance of doped Ta_3_N_5_. Meanwhile, Xiao et al. introduced gradient doping with Mg dopants in Ta_3_N_5_ for the first time instead of homogeneous doping [207]. As the Mg content increases, the conduction and valence bands of Mg:Ta_3_N_5_ lower. The gradient structure promotes charge separation efficiency by drifting photogenerated holes toward the semiconductor‐electrolyte interface and electrons toward the back (Figure 13E). As a result, the NiCoFe‐B_i_/gradient Mg:Ta_3_N_5_/Nb demonstrated a high photocurrent density of 8.5 mA cm^−2^ at 1.23 V versus RHE, compared to 7.5 mA cm^−2^ of homogeneous Mg:Ta_3_N_5_. In a subsequent study, the structure of La:Ta_3_N_5_/gradient Mg:Ta_3_N_5_ was designed considering the advantage of La doping in increased light absorption and the disadvantage of low bulk transport ability. This heterogeneous doping strategy further improved performance, demonstrating a photocurrent density of 10.06 mA cm^−2^ at 1.23 V versus RHE with the modification of a NiCoFe‐B_i_ co‐catalyst [204].

Advances in charge transport layers, protection layers, and co‐catalysts have also contributed to the improvement of Ta_3_N_5_ performance [30, 193, 208]. These functional overlayers and co‐catalysts not only increased the photocurrent density but also complemented the instability of Ta_3_N_5_, which self‐oxidizes rapidly. These multifaceted developments ultimately enable expanded applications of Ta_3_N_5_ photoanodes for unbiased solar water splitting. For example, Higashi et al. synthesized a Ta_3_N_5_ photoanode on a transparent GaN/Al_2_O_3_ substrate via high‐power sputtering and nitridation, attaining a transmittance of approximately 70% [209]. This transparent Ta_3_N_5_ photoanode enables the construction of bias‐free PEC‐photovoltaic (PV) tandem cells by integrating a CuInSe_2_ PV on the rear side (Figure 13F). In tandem cells, a current density of 7.3 mA cm^−2^ was achieved at the intersection of the photoanode and PV, resulting in a solar‐to‐hydrogen (STH) conversion efficiency of 9% (Figure 13G). The advancement in a tandem system illustrates the broader utilization of the Ta_3_N_5_ photoanode in efficient and sustainable solar water‐splitting systems.

#### 4.2.3 III‐Nitrides

III‐nitrides, including GaN (*E*_g_ = 3.4 eV), InN (*E*_g_ = 0.7 eV), and their alloys, have emerged as promising photoelectrocatalytic semiconductors. The band gap of III‐nitrides is tunable from 0.7 to 6.2 eV depending on the alloy composition. Hence, it can be adjusted to cover nearly the entire solar spectrum, making it suitable for various electrochemical reactions. Moreover, *n*‐type or *p*‐type doping provides favorable energy band bending suitable for redox reactions. In addition to their function as a light absorber, III‐nitrides have been applied as functional layers of other light absorbers with their charge transport ability and catalytic activity. Functionalities from polarity, crystal orientation, and surface‐terminated species contributed to an efficient and durable PEC pathway.

**FIGURE 14:**
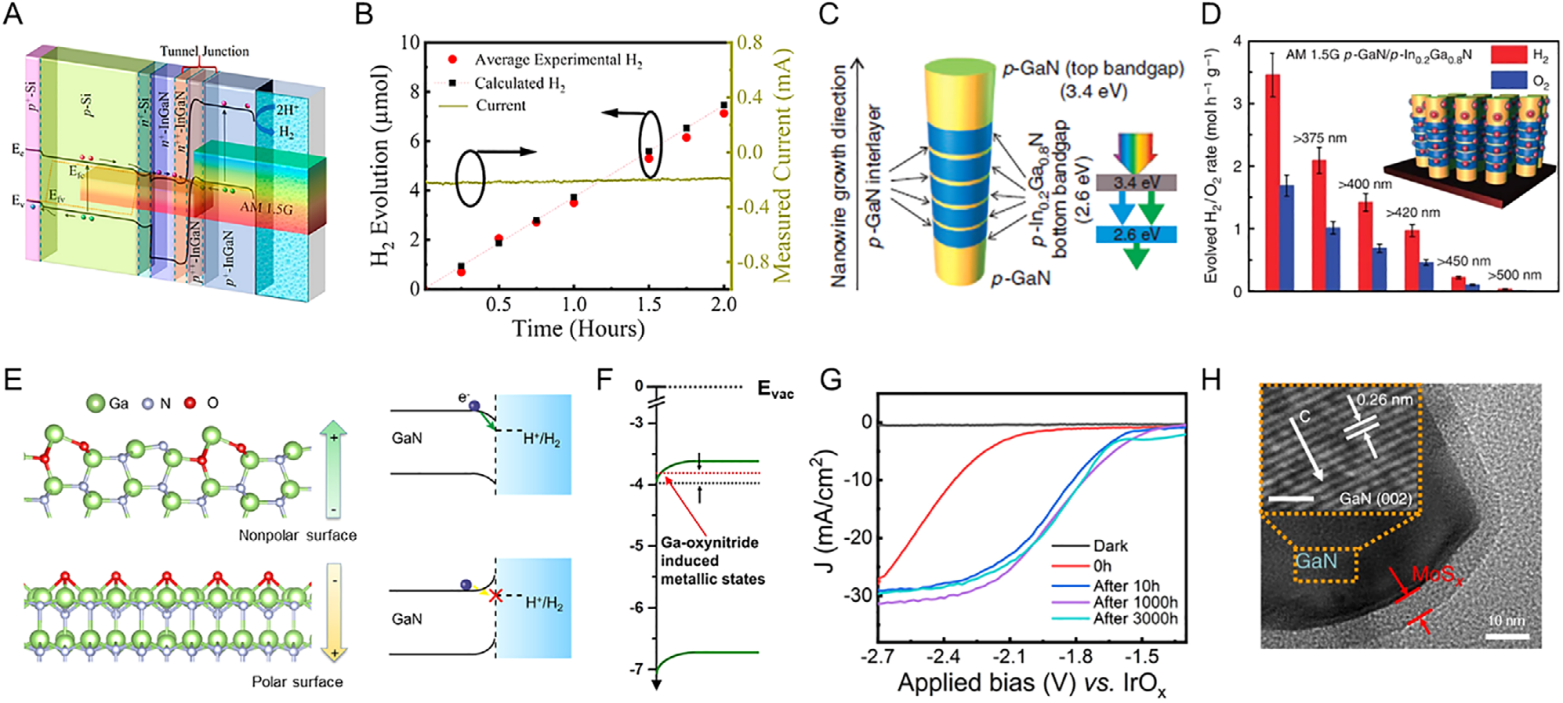
(A) Band‐diagram of the *p^+^*‐InGaN/TJ/*n^+^p*‐Si photocathode showing charge carrier generation in Si and *p^+^*‐InGaN, and charge extraction from *p^+^*‐InGaN. (B) H_2_ generation for sample B at 0 V versus IrO*_x_* under AM 1.5G illumination in 0.5 M H_2_SO_4_. Reprinted with permission [212]. Copyright 2020, American Chemical Society. (C) Schematic of the double‐band GaN/In_0.20_Ga_0.8_N nanowire heterostructure illustrating different layers incorporated during growth for efficient photon absorption and water‐splitting reaction. (D) H_2_ and O_2_ evolution rates in overall water splitting with AM1.5G illumination (≈26 suns), and with different long‐pass filters. Inset: Schematic of core/shell Rh/Cr_2_O_3_ nanoparticle decorated double‐band p‐GaN/p‐In_0.20_Ga_0.8_N nanowire photocatalyst on Si substrate. Reprinted with permission [215]. Copyright 2015, Nature Publishing Group. (E) Schematic illustrations of DFT configurations for surface polarization calculation of different GaN surfaces and the surface band bending variation induced by oxygen substitution and adsorption. Reprinted with permission [217]. Copyright 2022, American Chemical Society. (F) Band diagram of GaN with Ga‐oxynitride‐induced metallic states. (G) *J*–*V* curves of *n* ^+^‐GaN nanowires/Si photocathode at 0 h, after 10 h, after 1000 h, and after 3000 h in 0.5 M H_2_SO_4_ under AM 1.5 G illumination. Reprinted with permission [218]. Copyright 2023, Nature Publishing Group. (H) TEM image of MoS_x_@GaN nanowire. Inset: HR‐TEM image of the GaN core of MoS*_x_* @GaN nanowire. Reprinted with permission [220]. Copyright 2018, Nature Publishing Group.

Since GaN alone has an absorption range limited to the UV region, photoelectrodes using InGaN as a light absorber have been developed [210]. Chu et al. synthesized a highly crystalline In_0.5_Ga_0.5_N photoanode with a bandgap of 1.7 eV using molecular beam epitaxy [211]. The synthesized In_0.5_Ga_0.5_N photoanode with an IrO*_x_* co‐catalyst showed a fast onset potential of 0.1 V versus RHE and a photocurrent density of 10.9 mA cm^−2^ at 1.23 V versus RHE. Despite its small band gap of 1.7 eV, the low onset potential was the result of maximizing the advantages of exquisite band position. The band energetics of InGaN can also be effectively utilized as a top absorber in a monolithically integrated configuration. For instance, the integration of an *n* ^+^ *p*‐Si bottom light absorber with a band gap of 1.1 eV and a *p* ^+^‐InGaN top light absorber with a 2.2 eV bandgap fully exploits a significant portion of the solar spectrum (Figure 14A). The monolithic photocathode combines two light absorbers via a *p* ^++^‐InGaN/*n* ^++^‐InGaN tunnel junction (TJ). In this configuration, photogenerated electrons from *n* ^+^ *p*‐Si migrate to the *n* ^+^‐InGaN nanowire arrays, while those from *p* ^+^‐InGaN move toward the electrolyte. At the TJ, electrons from the bottom *n* ^+^ *p*‐Si and holes from the top *p* ^+^‐InGaN recombine. The TJ was formed on *n* ^+^‐InGaN to reduce defects and dislocations, playing a key role in connecting the two subcells. Consequently, the *p* ^+^‐InGaN/*n* ^+^ *p*‐Si photocathode modified with Al_2_O_3_/Pt validated the photocurrent and H_2_ evolution at 0 V versus IrO*_x_* in an acidic electrolyte (Figure 14B) [212]. Meanwhile, III‐nitrides that straddle the potentials for both OER and HER enable the development of wireless overall water‐splitting systems in the form of photoelectrodes. GaN nanowire arrays initially demonstrated the simultaneous evolution of H_2_ and O_2_ under light illumination [213, 214]. The subsequent introduction of heterojunctions with GaN and InGaN substantially increased hydrogen production compared to single GaN devices. Kibria et al. designed a multi‐stacked structure of *p*‐GaN/*p*‐In_0.2_Ga_0.8_N nanowires, aligning InGaN along GaN's growth direction to minimize non‐radiative recombination induced by misfit dislocations (Figure 14C) [215]. The *p*‐GaN/*p*‐In_0.2_Ga_0.8_N nanowires with Rh/Cr_2_O_3_ showed H_2_ and O_2_ generation rates of ≈3.46 mol h^−1^ g^−1^ and −1.69 mol h^−1^ g^−1^, respectively, and an STH efficiency of 1.8% under light illumination (≈26 suns) (Figure 14D). Recently, GaN/InGaN nanowires have been reported to achieve an STH efficiency of up to 9.2% in pure water through temperature‐dependent performance optimization [216]. With advancements in III‐nitrides, solar hydrogen production is becoming a more practical approach.

Among III‐nitrides, GaN has been applied to other primary light absorbers to improve the transport, separation, and injection of photogenerated charges. GaN has a Wurtzite structure, where Ga and N atomic layers alternate, resulting in varying polarity depending on the crystal orientation due to the directional nature of Ga‐N bonds. Xiao et al. investigated the behavior of photogenerated charge carriers based on the polarity of GaN's crystal facets using *p*‐Si as a primary light absorber [217]. PEC activity was significantly superior in the nonpolar samples with exposed *m*‐plane compared to polar samples with exposed *c*‐plane. DFT calculations indicated that stable oxygen substitution and adsorption states induced positive and negative polarization in the nonpolar and polar planes, respectively (Figure 14E). Unlike the upward band bending observed on the polar surface, the downward band bending on the nonpolar surface facilitated charge transport, leading to the HER. For *m*‐plane GaN, it was subsequently discovered that the N‐terminated GaN surface exhibited an in‐situ self‐improvement, forming catalytically active GaON species. The locally formed oxynitride species were metallic, and this metallic surface state triggered downward band bending, further promoting the reduction reaction (Figure 14F). As a result, activated GaN/*n* ^+^ *p*‐Si showed little degradation even after a chronoamperometry test of 3000 h under 1 sun illumination in 0.5 M H_2_SO_4_ with 0.2 mM Triton X‐100, resulting in conversion to a stable hydrogen production surface without a co‐catalyst (Figure 14G) [218]. Furthermore, the performance of the Si photocathode was enhanced by the synergistic effect between the GaN nanowires/Si structure and the HER catalyst materials. At the interface between the Pt nanocluster and GaN, Pt‐Ga sites promoted water dissociation, ensuring HER operation stability even in seawater [219]. Also, the MoS*_x_*/GaN nanowires/Si structure improved electron transport ability from the geometric matching of GaN and MoS*_x_*, resulting in a high photocurrent density of 40 mA cm^−2^ at 0 V versus RHE in an acidic electrolyte (Figure 14H) [220]. In this way, GaN provides various functionalities through a deep understanding of interactions in the operating environment.

## 5 Conclusion and Perspective

Herein, we have systematically explored fundamental features and catalytic advantages and discussed the advances in synthetic processes and catalytic applications of metal nitride (photo)electrocatalysts. Metal nitrides exhibiting unique electronic structures and high electrical conductivity made them highly promising candidates for electrocatalysis. Especially in terms of electrocatalysis, the catalytic activity of metal nitrides was controlled by the delocalization of electrons according to the nitrogen content and the *d*‐band center of metal. In addition, defects due to nitrogen vacancies or heteroatom doping in metal nitride, as well as interfaces in the heterostructure, provide active sites on the surface, serving advantages for various electrochemical reactions. From a photoelectrochemical perspective, metal nitrides with suitable band gaps for light absorption were utilized as light absorbers, and when they formed appropriate band structures with other photoelectrodes, they were applied as a functional layer that promotes charge transport. According to the origin of the nitrogen atom that forms a bond with the host metal, various synthesis strategies, including direct ammonolysis, indirect ammonolysis, solution process, and vapor deposition, have been introduced to fabricate metal nitride catalysts. Based on the advantages of metal nitrides, such as high electrical conductivity, abundant active sites, and unique electronic and optical behaviors, they have exhibited superior catalytic activities in various electrochemical reactions. Metal nitrides have been extensively explored in HER, OER, ORR, NRR, CRR, and biomass valorization, successfully overcoming major challenges in electrocatalysis. Additionally, in photoelectrocatalysis, metal nitrides have shown great potential as light absorbers and functional layers, further broadening their application spectrum. Looking forward, several perspectives can be considered for the future development of metal nitride catalysts to fully exploit their potential.

First, it is important to develop advanced synthesis techniques to maximize the intrinsic properties of metal nitrides. Developing synthesis methods that allow for precise control over the morphology and size of metal nitrides is crucial. In particular, it is necessary to obtain the advantages of maximized specific surface area and active sites by the solution process‐based nanostructures with great structural diversity and scalability. The synthetic approach should also be focused on identifying optimal dopants and alloy composition to enhance catalytic activity and scalability. Furthermore, scalable and environmentally friendly synthesis should be considered while maintaining cost‐effectiveness. In order to obtain the desired phase, stoichiometry, and shape of metal nitrides, a complex and high‐temperature synthesis process is required. The difficulty in controlling these synthesis processes and producing them on a large scale is considered a limitation of metal nitrides. Current nitriding processes based on the reaction involving high‐purity ammonia and high temperature lower the scalability and cost‐effectiveness. The nitridation barrier must be dramatically lowered by exploring non‐toxic but active nitrogen sources.

Second, metal nitrides should expand into hybrid and composite materials. Combining metal nitrides with carbon‐based materials, metal carbides, or metal oxides can create hybrid structures that exhibit synergistic catalytic effects. Based on heterointerfaces, these composites can increase active site exposure and improve charge transfer, enhancing overall catalytic performances. Metal nitrides have the disadvantage of being particularly susceptible to self‐oxidation during electrochemical reactions. The instability issue of metal nitrides derived from the self‐oxidation reaction during electrocatalysis could be improved by the hybrid and composite materials. When creating core‐shell structures or embedding metal nitrides into the robust matrix, the hybrid structures enhance the structural stability of metal nitrides by preventing aggregation or degradation during catalytic reactions. Additionally, designing multifunctional catalysts based on hybrid materials is a promising approach. By designing bifunctional catalysts for both oxidation and reduction reactions, the efficiency of electrochemical devices is significantly improved by reducing the need for separate catalysts for each reaction. Especially, single‐atom catalysts have demonstrated exceptional performance in the field of catalysis due to their unique properties, such as maximized atomic efficiency, tunable electronic structures, and highly active sites [221]. Given the promising properties of single‐atom catalysts, the potential of metal nitride‐supported single‐atom catalysts is noteworthy. Metal nitrides themselves possess excellent properties, such as high stability, electrical conductivity, and chemical resistance. When combined with single atoms, metal nitrides could serve as effective supports, enhancing the catalytic performance by providing a robust platform that stabilizes the single atoms and facilitates their interaction with reactants.

Third, designing metal nitrides must be accompanied by theoretical and computational approaches. The computational method, such as DFT calculations, can provide information about the electronic structure of metal nitrides, helping understand the relationship with catalytic activity. Also, theoretical studies can elucidate the reaction mechanisms of various catalytic reactions at the atomic level, identifying key intermediates and rate‐determining steps. This knowledge can offer insight into the rational design of catalysts with improved kinetics and stability. Utilizing machine learning can accelerate the discovery of new metal nitride catalysts. By rapidly screening a large number of potential nitrides, promising candidates for experimental validation can be identified. In addition to electrocatalysis, this approach will be an important tool for discovering new light absorbers in photoelectrolysis.

Finally, metal nitrides should be expanded to a wider range of catalytic reactions. Metal nitrides have been applied to simple catalytic reactions such as HER, OER, and ORR. Exploring new electrocatalysis beyond the current scope can unlock the full potential of metal nitrides in various fields. In nitrogen and nitrate reduction, as well as ammonia and urea oxidation, where nitrogen atoms are included in the reaction step, nitrogen atoms on the surface of the nitride electrode are involved in the reaction, providing a promising mechanism beyond that for metal or metal oxide‐based catalysts. Utilizing metal nitrides for biomass valorizations, which overcome the sluggish kinetics of OER and produce valuable products, is also a promising approach. Over the past decades, comprehensive efforts have been made based on metal nitrides in (photo)electrocatalysis. Based on these, more advanced catalytic reactions using metal nitrides can be achieved by reflecting the aforementioned challenges of design, synthesis, calculation, and application.

## Conflicts of Interest

The authors declare no conflict of interest. Ho Won Jang is a member of the *Exploration* editorial board, and he was not involved in the handling or peer review process of this manuscript.
